# Platelet‐Derived Growth Factor C Facilitates Malignant Behavior of Pancreatic Ductal Adenocarcinoma by Regulating SREBP1 Mediated Lipid Metabolism

**DOI:** 10.1002/advs.202407069

**Published:** 2024-09-03

**Authors:** Yin‐Hao Shi, Zhi‐De Liu, Ming‐Jian Ma, Guang‐Yin Zhao, Ying‐Qin Zhu, Jie‐Qin Wang, Yang‐Yin‐Hui Yu, Xi‐Tai Huang, Jing‐Yuan Ye, Fu‐Xi Li, Xi‐Yu Wang, Qiong‐Cong Xu, Xiao‐Yu Yin

**Affiliations:** ^1^ Department of Pancreato‐Biliary Surgery the First Affiliated Hospital of Sun Yat‐sen University Guangzhou 510080 China; ^2^ Animal Experiment Center of the First Affiliated Hospital of Sun Yat‐sen University Guangzhou 510080 China; ^3^ Department of Pediatric Surgery Guangzhou Women and Children's Medical Center Guangzhou Medical University Guangzhou 510623 China; ^4^ Guangdong Provincial People's Hospital Guangdong Academy of Medical Sciences Guangzhou 510080 China

**Keywords:** lipid metabolism, metastasis, PDAC, PDGFC, SREBP1

## Abstract

Lipid metabolism reprogramming stands as a fundamental hallmark of cancer cells. Unraveling the core regulators of lipid biosynthesis holds the potential to find promising therapeutic targets in pancreatic ductal adenocarcinoma (PDAC). Here, it is demonstrated that platelet‐derived growth factor C (PDGFC) orchestrated lipid metabolism, thereby facilitated the malignant progression of PDAC. Expression of PDGFC is upregulated in PDAC cohorts and is corelated with a poor prognosis. Aberrantly high expression of PDGFC promoted proliferation and metastasis of PDAC both in vitro and in vivo. Mechanistically, PDGFC accelerated the malignant progression of PDAC by upregulating fatty acid accumulation through sterol regulatory element‐binding protein 1 (SREBP1), a key transcription factor in lipid metabolism. Remarkably, Betulin, an inhibitor of SREBP1, demonstrated the capability to inhibit proliferation and metastasis of PDAC cell lines, along with attenuating the process of liver metastasis in vivo. Overall, the study underscores the pivotal role of PDGFC‐mediated lipid metabolism in PDAC progression, suggesting PDGFC as a potential biomarker for PDAC metastasis. Targeting PDGFC‐induced lipid metabolism emerges as a promising therapeutic strategy for metastatic PDAC, with the potential to improve clinical outcomes.

## Graphical abstract

Platelet‐derived growth factor C (PDGFC) enhances fatty acid‐associated lipid metabolism, accelerating proliferation and metastasis, and correlating with poor prognosis in PDAC. Its upregulation promotes malignant behavior through SREBP1, a critical transcription factor in lipid metabolism. Targeting PDGFC‐induced lipid metabolism is a promising therapeutic strategy for metastatic PDAC, holding promise for improved clinical outcomes.

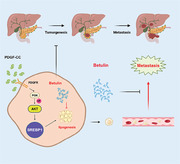

## 1 Introduction

Pancreatic ductal adenocarcinoma (PDAC) is a devastating disease characterized by rapid progression and a poor prognosis, largely attributed to late detection. Given its remarkable resistance to conventional chemotherapy and molecule‐targeted therapy, it is highly susceptible to recurrence and metastasis, resulting in an overall five‐year survival rate of less than 5%.^[^ ^1^ ^]^ In recent years, the global incidence and mortality rate of PDAC have continued to rise, with over half of PDAC patients presenting with distant metastasis at the time of diagnosis. Even for those who have the opportunity for curative resection, ≈80% will experience relapse within two years.^[^ ^2^ ^]^ Given the critical challenges in PDAC treatment, there is a pressing need for further exploration of sensitive and specific metastatic markers, as well as the development of novel anti‐tumor strategies for PDAC.

Dysregulated growth factor signaling is crucial for the uncontrolled proliferation and division of cancer cells. Similarly, the metastasis of PDAC is a complex process in which growth factors play a significant role.^[^ ^3^ ^]^ Growth factors are a type of peptide substances that regulate cell growth or function by binding to specific cell membrane receptors. Studies have shown that the expression and function of cytokines, including growth factors and chemotactic factors, change significantly in malignant tissues compared with normal tissues.^[^ ^4^ ^]^ The platelet‐derived growth factor family is involved in the onset and development of various tumors including non‐small‐cell lung cancer, gastrointestinal stromal tumors, breast cancer, ovarian cancer, and liver cancer.^[^ ^5^ ^]^ The vascular endothelial growth factor (VEGF) family is commonly overexpressed in the majority of human tumors and is associated with invasion, vascular density, metastasis, recurrence, and prognosis.^[^ ^6^ ^]^ Furthermore, epidermal growth factors (EGF) and transforming growth factor (TGF) family also play crucial roles in the process of carcinogenesis.^[^ ^7^ ^]^

Among them, platelet‐derived growth factor (PDGF) family is one of the most widely studied growth factors and PDGFC is a member of this family. Previous studies have shown that PDGFC plays a vital role in embryonic development and adult physiological processes, mainly mediating the formation of ducts or duct‐like structure. PDGFC may be related to the occurrence and development of various malignant tumors. In colorectal cancer, PDGFC serves as a tumorigenic factor, playing a role in metastasis and tumor grading.^[^ ^8^ ^]^ In triple‐negative breast cancer, the expression of PDGFC is significantly higher than that of PDGFA, PDGFB, and PDGFD. Simultaneously, the abnormally elevated expression of PDGFC is related to a poor prognosis for patients. Moreover, the platelet‐derived growth factor receptor (PDGFR) inhibitors sunitinib and ponatinib significantly suppress the proliferation and metastasis of triple‐negative breast cancer cell lines.^[^ ^9^ ^]^ In malignant melanoma, PDGFC dimers promote tumor growth in a paracrine manner by recruiting and activating cancer‐associated fibroblasts (CAFs).^[^ ^10^ ^]^ These studies have indicated that PDGFC may be involved in the malignant progression of various tumors, but the specific cancer‐promoting mechanisms are quite different.

In recent years, lipid metabolic reprogramming has become one of the most prominent research fields in tumors. In addition to providing energy, lipids also contribute to the formation of cell membranes and serve as precursors for signaling molecules.^[^ ^11^ ^]^ Fatty acid metabolism, as a crucial component of lipid metabolism, is not only important for the growth and development of normal cells but also plays a crucial role in the initiation, progression, and metastasis of tumors. Compared to normal cells, tumor cells exhibit not only faster proliferation and increased energy requirements but also a close association between energy production and substance metabolism.^[^ ^12^ ^]^ Sterol regulatory element binding protein (SREBP) is a member of the transcription factors family that regulates the expression of genes crucial for the uptake and synthesis of fatty acids, cholesterol, and phospholipids.^[^ ^13^ ^]^ Beyond its role in mediating lipid biosynthesis, SREBP1 is also associated with tumor progression and metastasis.^[^ ^14^ ^]^ While current studies have indicated the involvement of lipid metabolism and growth factor dysfunction in the occurrence and development of PDAC,^[^ ^15^ ^]^ the underlying mechanism remains to be elucidated.

Here, we delved into the role of PDGFC in the malignant progression of PDAC. Our findings reveal that PDGFC, an important member of the growth factor family, is closely related to PDAC metastasis. Elevated expression of PDGFC is positively correlated with the poor prognosis of PDAC patients. Further, we found that PDGFC promotes the proliferation, invasion, and migration of PDAC in vitro and in vivo. Mechanistically, we found that PDGFC promotes the expression of the key lipid metabolic protein SREBP1, thereby mediating fatty acid metabolism. Betulin, a lipid metabolism inhibitor, demonstrates effective attenuation of the malignant progression of PDAC. In summary, PDGFC emerges as a novel upstream signal that promotes lipid biosynthesis, thereby facilitating the malignant behavior of PDAC. Inhibiting PDGFC‐related lipid biosynthesis in PDAC proves to be an effective strategy for alleviating liver metastasis.

## 2 Results

### 2.1 PDGFC Stands as a Potential Predictor of Proliferation and Metastasis in PDAC

**Figure 1:**
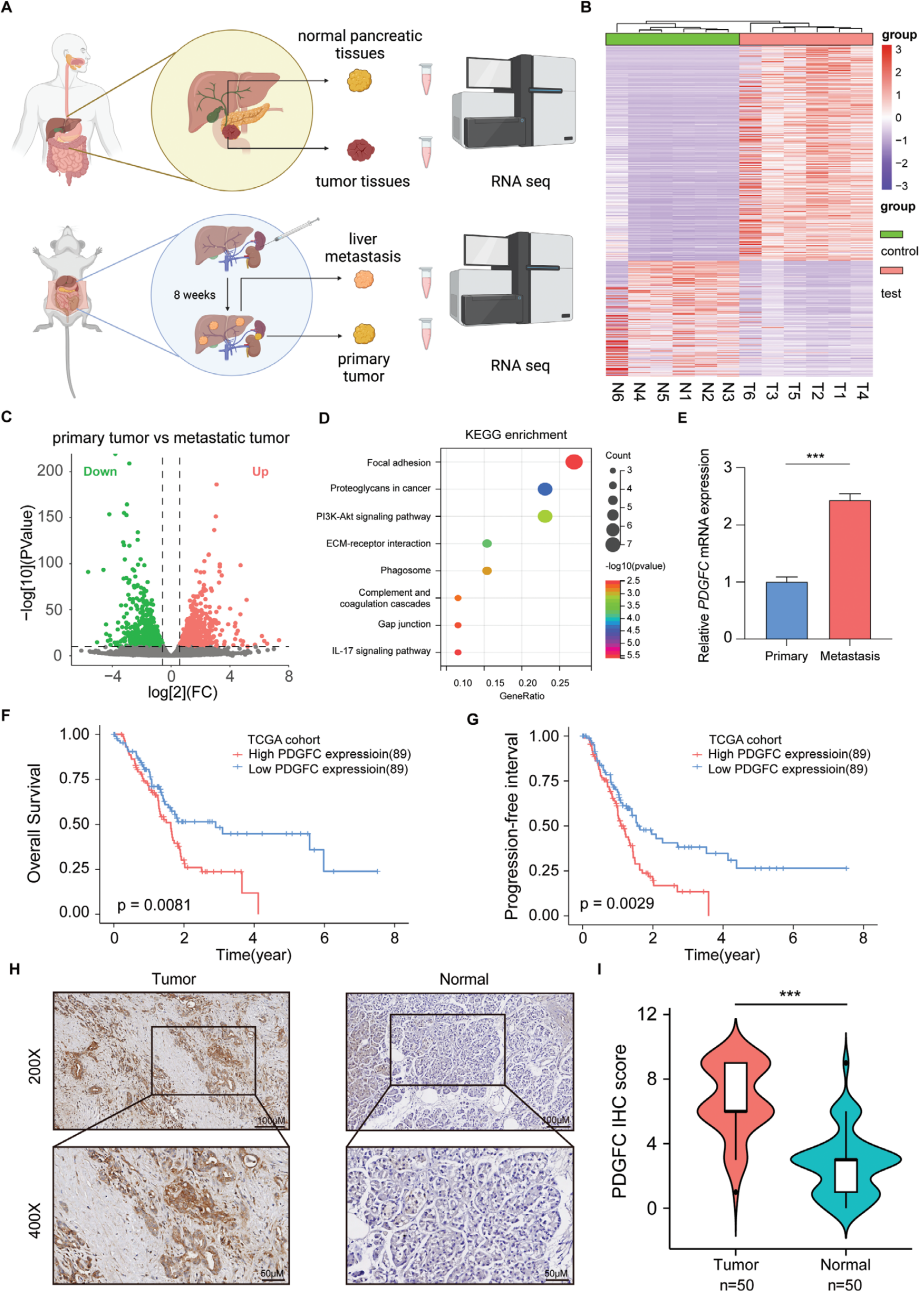
PDGFC stands as a potential predictor of proliferation and metastasis in PDAC. A) Schematic diagram of RNA sequencing in PDAC tumor tissues and liver metastatic tumors created with BioRender.com. B) Heatmap of the DEGs in RNA sequencing results of 6 pairs of pancreatic tumor tissues and adjacent tumor tissues. C) Volcano plot of DEGs in RNA sequencing results of primary tumors and liver metastatic tumors. D) KEGG enrichment results of commonly upregulated DEGs in pancreatic tumor tissues and liver metastasis tissues. E) The mRNA levels of PDGFC are significantly elevated in liver metastatic tumors compared to primary tumors. F) High expression of PDGFC is negatively correlated with the overall survival of PDAC patients from TCGA dataset. G) High expression of PDGFC is negatively correlated with the progression‐free interval of PDAC patients from TCGA dataset. H) Representative images of immunohistochemical (IHC) staining results for 50 pairs of pancreatic tumor tissues and normal tissues. I) Statistical results of IHC staining for 50 pairs of pancreatic tumor tissues and normal tissues. Data are presented as mean ± SD (n = 3 in E) ^***^ *p* < 0.001 according to Student's *t*‐test.

In our quest to identify potential predictors of proliferation and metastasis in PDAC, we collected fresh samples of tumor and normal tissue from six patients with PDAC who underwent surgical resection at our center. In addition, we successfully constructed a mouse model of liver metastasis in PDAC and collected tissue samples from primary tumors and liver metastases. Subsequently, these samples were sent for RNA sequencing to undergo further analysis (**Figure** **1**A). We initially selected genes that were upregulated in six pairs of tumor tissues compared to adjacent tumor tissues, as well as genes upregulated in liver metastasis compared to primary tumors (Figure 1B–C; Figure S1A. Supporting Information). The common upregulated 42 genes in these two sets of genes suggest their association with the proliferation and metastasis of PDAC (Figure S1B, Supporting Information). Next, we conducted KEGG enrichment on these 42 genes and found that most of them are related to signaling pathways such as focal adhesion, proteoglycans in cancer, and PI3K/AKT signaling pathway (Figure 1D). To further narrow down the scope of our study, we incorporated three GEO datasets and performed a combined analysis of the upregulated differentially expressed genes (DEGs) with focal adhesion‐related genes. The result suggested that PDGFC may be a key factor in the proliferation and metastasis of PDAC (Figure S1C,D, Supporting Information). Moreover, we observed a significant upregulation of PDGFC expression in both tumor tissues and liver metastasis (Figure 1E; Figure S1E, Supporting Information). Given that PDGFC is a member of the PDGF family, we further explored other molecules in this family. The sequencing data showed that the expression of PDGFC was significantly increased in PDAC tumor tissues (Figure S1F–H, Supporting Information). In addition, the abnormal high expression of PDGFC is positively correlated with poor prognosis in PDAC patients (Figure 1F,G). The protein level of PDGFC was also higher than that in normal pancreatic tissues in 50 cases of PDAC tissues from our center (Figure 1H–I). These findings suggest that PDGFC holds promise as a potential predictive marker for tumor proliferation and metastasis in PDAC patients.

### 2.2 PDGFC Promotes PDAC Progression In Vitro

**Figure 2:**
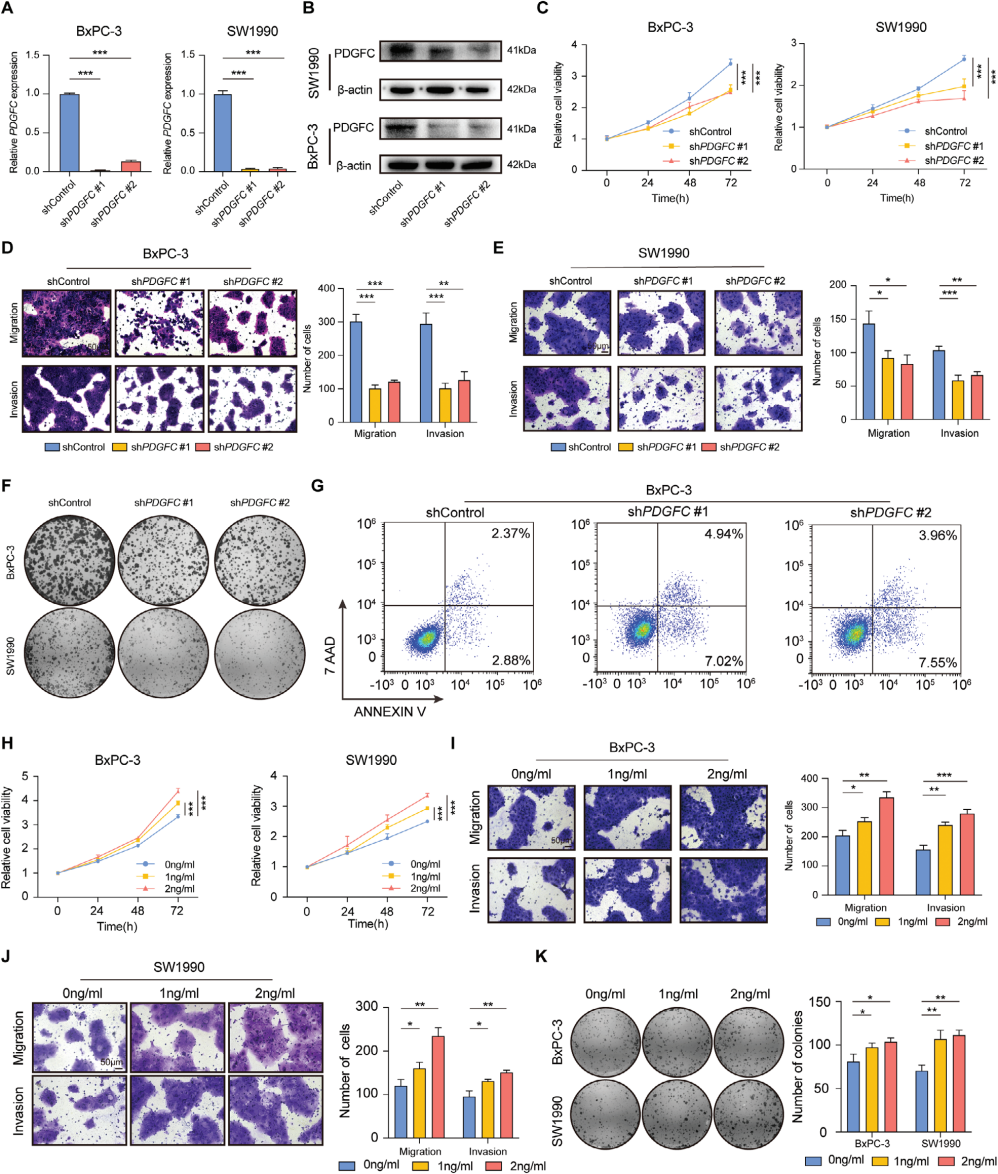
PDGFC promotes PDAC progression in vitro. A) The mRNA level of BxPC‐3 and SW1990 cells after PDGFC silencing confirmed by RT‐qPCR. B) The protein level of BxPC‐3 and SW1990 cells after PDGFC silencing confirmed by western blotting. C) Cell growth curve of BxPC‐3 and SW1990 cells transfected with PDGFC shRNA or shControl. D) Cell migration and invasion ability of BxPC‐3 cells after shPDGFC transfection. E) Cell migration and invasion ability of SW1990 cells after shPDGFC transfection. F) Colony‐forming assays of PDAC cells after PDGFC silencing. G) Apoptotic assays of BxPC‐3 cells after transfected with shPDGFC or shControl. H) Cell growth curve of BxPC‐3 and SW1990 cells treated different doses of human recombinant PDGFC protein. I) Cell migration and invasion ability treated with different doses of human recombinant PDGFC protein in BxPC‐3 cells. J) Cell migration and invasion ability treated with different doses of human recombinant PDGFC protein in SW1990 cells. K) Colony‐forming assays of BxPC‐3 and SW1990 cells treated with different doses of human recombinant PDGFC protein. Data are presented as mean ± SD (n = 3). ^*^ *p* < 0.05; ^**^ *p* < 0.01; ^***^ *p* < 0.001 according to Student's *t*‐test.

To explore the biological role of PDGFC in the malignant progression of PDAC, stable PDGFC‐knockdown PDAC cells were established. The knockdown efficiency of PDGFC was confirmed on mRNA and protein levels (**Figure** **2**A,B). After PDGFC silencing, there was a significant decrease in the proliferation rate of PDAC cells (Figure 2C). As expected, the migration, invasion, and colony formation ability of PDAC cells were also dramatically inhibited after PDGFC knockdown (Figure 2D–F; Figure S2A, Supporting Information). Furthermore, we observed that PDGFC knockdown increased the apoptotic rate of PDAC cells, indicating the anti‐tumor effect of PDGFC inhibition (Figure 2G; Figure S2B,C, Supporting Information). To validate the results above, we selected a potent inhibitor crenolanib (TargetMol, Shanghai, China) to alleviate PDGFC signal in vitro (Figure S3A, Supporting Information). Our results indicated that crenolanib effectively inhibited the proliferation and metastasis of PDAC cells, while increasing their apoptotic rates simultaneously (Figure S3B–D, Supporting Information).

Subsequently, we simulated the condition of PDGFC overexpression by introducing recombinant human PDGF‐CC protein (Abcam, Shanghai, China) into the culture medium. Under the stimulation of different doses of recombinant human PDGF‐CC protein, the proliferation, migration, and invasion ability of PDAC cells were mildly enhanced (Figure 2H–J). Colony formation assay also indicated improved colony‐forming ability after PDGFC overexpression (Figure 2K). These results suggest that PDGFC is essential in promoting PDAC progression.

### 2.3 Depletion of PDGFC Impairs the Malignant Behavior of PDAC In Vivo

**Figure 3:**
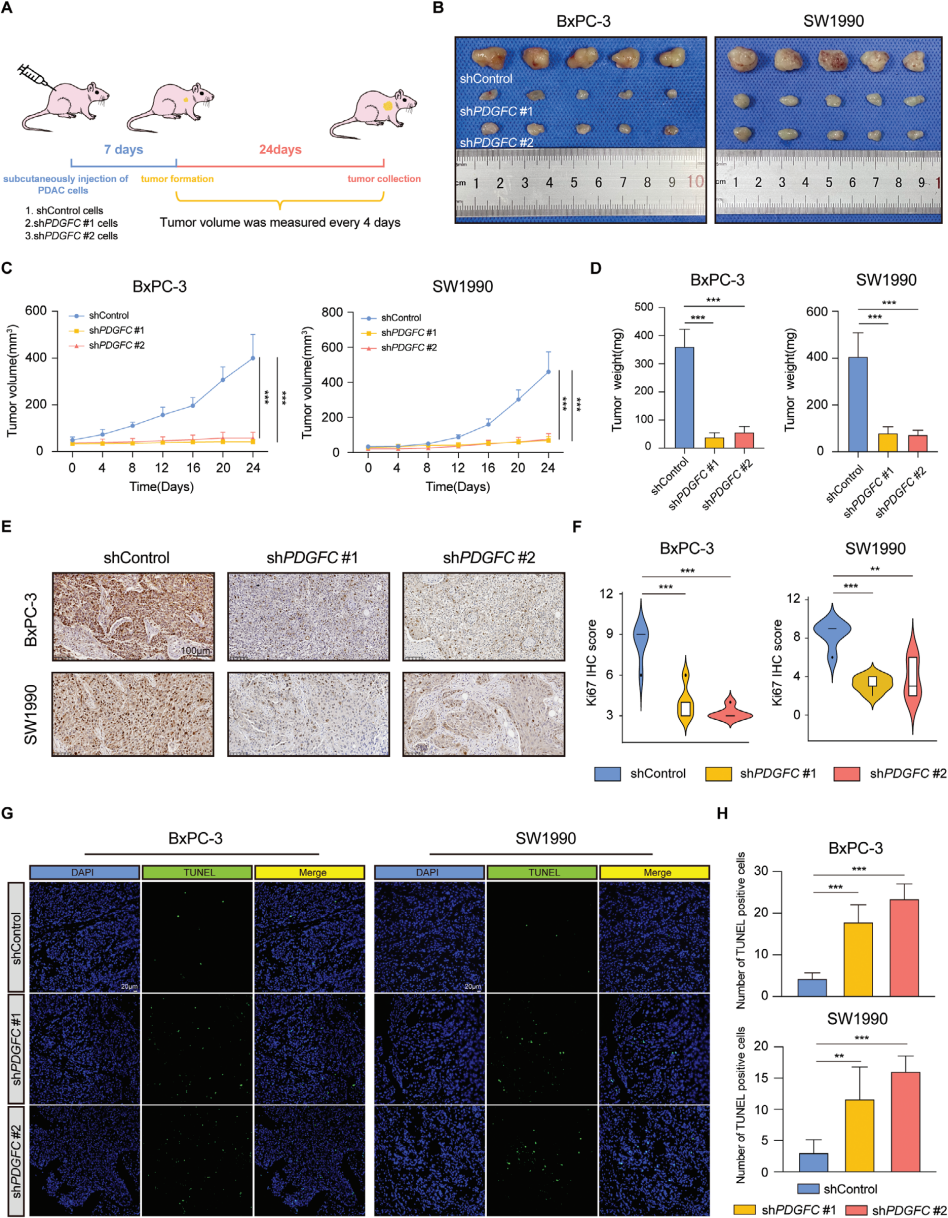
Depletion of PDGFC impairs the malignant behavior of PDAC in vivo. A) Schematic diagram of xenografts in BALB/c nude mice by inoculating PDAC cells transfected with shControl or shPDGFC at their right axillae. B) Xenograft tumors derived from BxPC‐3 and SW1990 cells were shown. C) Tumor growth curves after the injection of PDAC cells transfected with shControl or shPDGFC. D) Tumor weight of each group. E) Representative IHC staining of Ki67 in tumors from each group. F) Statistical analysis of IHC staining of Ki67 in tumors from each group. G) Representative images of TUNEL apoptotic assay from different group. H) Statistical analysis of TUNEL apoptotic assay from different group. Data are presented as mean ± SD (n = 5). ^**^ *p* < 0.01; ^***^ *p* < 0.001 according to Student's *t*‐test.

To comprehensively analyze the impact of PDGFC on PDAC progression in vivo, we subcutaneously injected stable PDGFC knockdown BxPC‐3 and SW1990 cells into BALB/c nude mice at their right axillae. After ≈ 7 days of feeding, the tumor volume was measured every 4 days (**Figure** **3**A). The PDGFC‐knockdown tumors developed more slowly than those in the control group (Figure 3B,C). Additionally, there was also a notable reduction in tumor weight after PDGFC silencing compared to the control group (Figure 3D). To delve further into the mechanistic basis of PDAC progression inhibition, we performed immunohistochemistry (IHC) staining for Ki67, an objective marker indicating cell proliferation status. The IHC results demonstrated a substantial reduction in Ki67 levels in PDGFC knockdown tumors compared to the control group (Figure 3E,F). In addition, the TUNEL assay revealed a higher number of apoptotic cells in the shPDGFC tumors than that in the control group (Figure 3G,H). Taken together, these findings offer compelling evidence that PDGFC knockdown substantially inhibits the malignant progression of PDAC, supporting its potential as a promising marker for predicting tumor progression.

### 2.4 Fatty Acid Metabolism is a Downstream Crucial Target of PDGFC in PDAC

**Figure 4:**
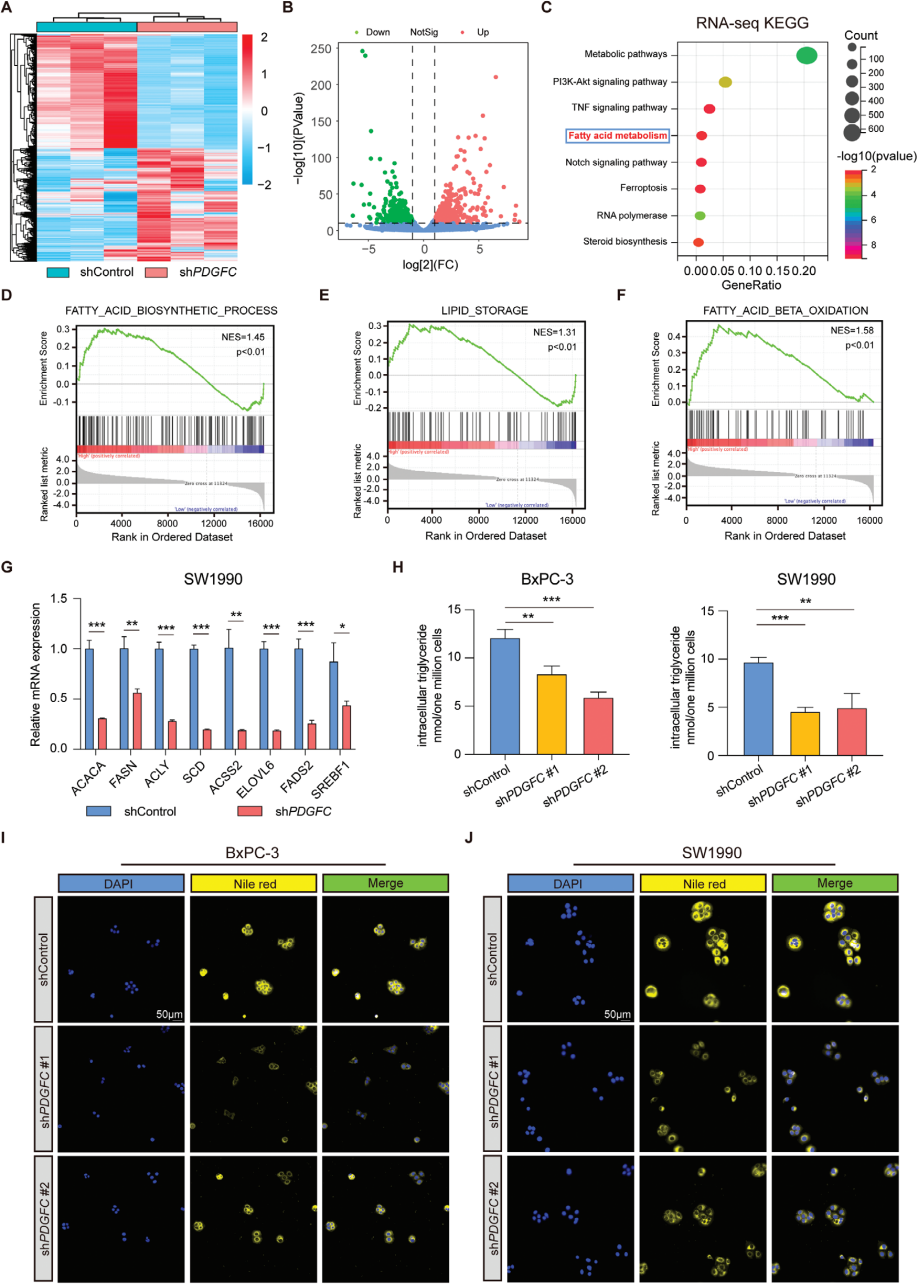
Fatty acid metabolism is a downstream crucial target of PDGFC in PDAC. A) Heatmap showing the expression changes between shControl and shPDGFC group in BxPC‐3 cells. B) Volcano plot showing the upregulated and downregulated genes after PDGFC silencing or not. C) KEGG enrichment of DEGs between shControl and shPDGFC cells. D) GSEA enrichment results suggest an association between PDGFC and fatty acid biosynthetic process. E) GSEA enrichment results suggest an association between PDGFC and lipid storage. F) GSEA enrichment results suggest an association between PDGFC and fatty acid beta‐oxidation. G) The mRNA levels of lipid synthesis‐relatedkey genes after PDGFC knockdown in SW1990 cells. H) The intracellular triglyceride level in BxPC‐3 and SW1990 cells transfected with PDGFC or shControl. I) Representative images of Nile red staining in BxPC‐3 cells transfected with PDGFC or shControl. J) Representative images of Nile red staining in SW1990 cells transfected with PDGFC or shControl. Data are presented as mean ± SD (n = 3 in G and H). ^*^ *p* < 0.05; ^**^ *p* < 0.01; ^***^ *p* < 0.001 according to Student's *t*‐test.

To delineate the potential molecular mechanism underlying the facilitation of PDAC malignant progression by PDGFC, we conducted RNA‐seq analysis in BxPC‐3 cells with or without PDGFC knockdown (**Figure** **4**A). The results revealed that 4336 transcripts were upregulated upon PDGFC knockdown, while 4243 transcripts were downregulated with statistical significance (Figure 4B). To identify the potential downstream target influenced by PDGFC, we performed KEGG enrichment on the DEGs. The results indicated significant enrichment of DEGs in pathways related to metabolism, particularly those associated with fatty acid metabolism (Figure 4C). Tumor cells often undergo metabolic alterations characterized by increased levels of circulating free fatty acids, monacylglycerides, and diacylglycerides.^[^ ^16^ ^]^ Therefore, we hypothesized that PDGFC might contribute to the malignant progression of PDAC through the regulation of fatty acid metabolism. Subsequently, GSEA enrichment analysis was conducted, revealing an association between PDGFC‐related DEGs and fatty acid biosynthesis. This association indicated an augmentation in intracellular lipid accumulation and enhanced utilization of fatty acids (Figure 4D–F). To validate this hypothesis, we observed a downregulation of key genes associated with lipid metabolism at the mRNA level following PDGFC knockdown (Figure 4G; Figure S4A, Supporting Information). Interestingly, the intracellular triglyceride levels exhibited a decrease after PDGFC silencing (Figure 4H). Moreover, Nile red staining illustrated a reduction in lipid droplets within PDAC cells in the shPDGFC group compared to the control group (Figure 4I,J). In addition, we simulated the aberrant expression of PDGFC using human PDGFC recombinant protein. The results demonstrated that the mRNA levels of genes related to lipid metabolism were upregulated after PDGFC stimulation (Figure S4B, Supporting Information). This upregulation led to a corresponding increase in the level of intracellular triglycerides (Figure S4C, Supporting Information), which was further confirmed by Nile red staining (Figure S4D,E, Supporting Information). These findings suggest that PDGFC promotes PDAC progression through the abnormal activation of lipid synthesis.

### 2.5 PDGFC Regulates SREBP1 via PI3K/AKT Signaling Pathway to Promote Lipid Biosynthesis

**Figure 5:**
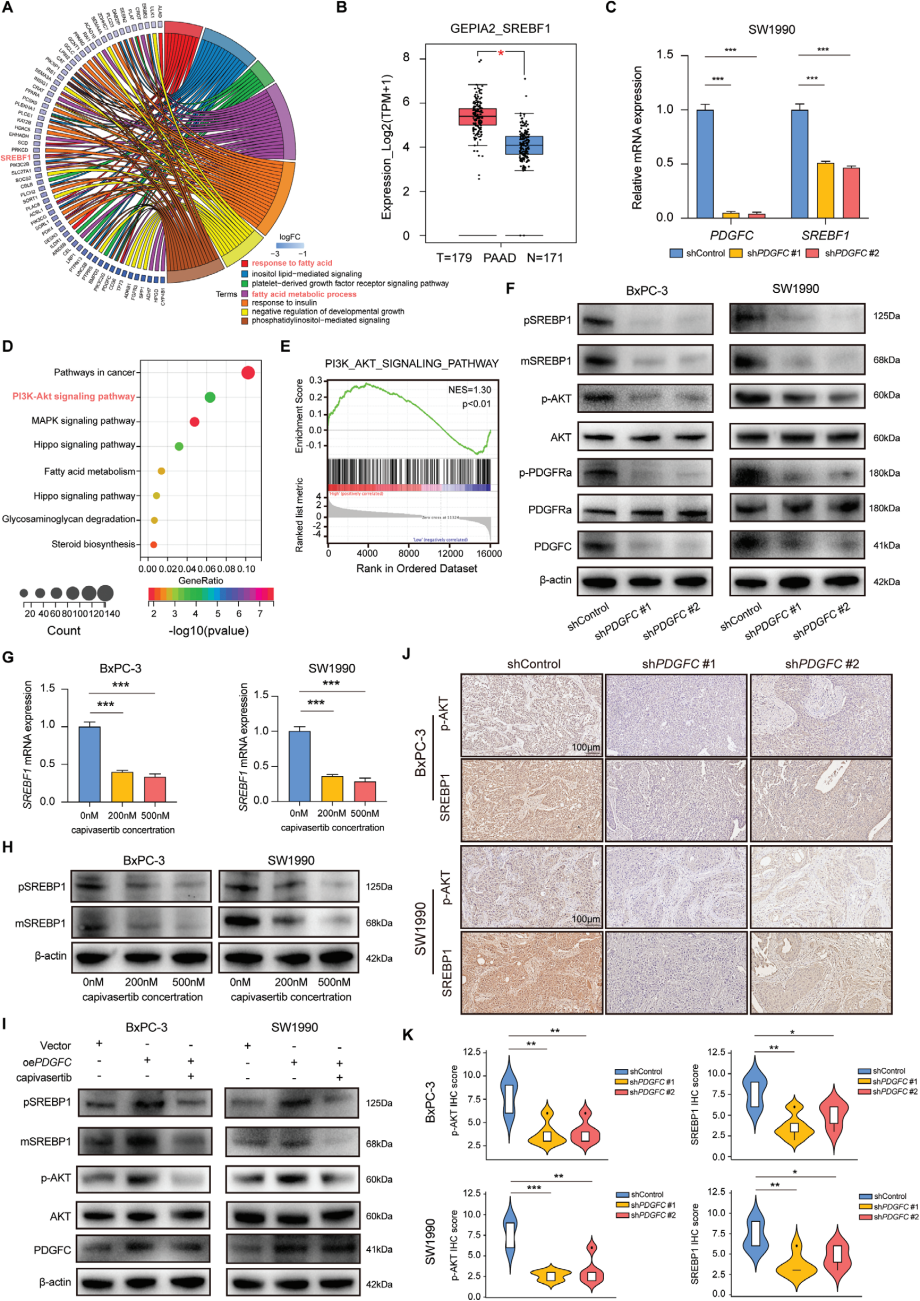
PDGFC regulates SREBP1 via PI3K/AKT signaling pathway to promote lipid biosynthesis. A) GO molecular function enrichment of DEGs in BxPC‐3 cells silencing PDGFC or not. B) The mRNA level of SREBF1 between PDAC tumor tissue or normal tissue in GEPIA2 database. C) The mRNA level of SREBF1 after PDGFC silencing in SW1990 cells. D) KEGG enrichment of DEGs after PDGFC silencing or not. E) GSEA enrichment results suggest PI3K/AKT signaling pathway is the downstream target of PDGFC. F) The protein level of PDGFR/PI3K/AKT signaling pathway and SREBP1 after PDGFC silencing were confirmed by western blotting. G) The mRNA level of SREBF1 after capivasertib treatment in PDAC cells. H) The protein level of SREBP1 after capivasertib treatment in PDAC cells. I) The protein level of PDGFC, AKT, p‐AKT, and SREBP1 with different treatment in PDAC cells. J) Representative images of IHC in xenograft tumors about p‐AKT and SREBP1. K) Statistical analysis of IHC staining. Data are presented as mean ± SD (n = 3 in C and G, n = 5 in J and K). ^*^ *p* < 0.05; ^**^ *p* < 0.01; ^***^ *p* < 0.001 according to Student's *t*‐test.

To further illustrate the specific mechanism of the regulation of fatty acid metabolism by PDGFC, we scrutinized the RNA‐seq data from BxPC‐3 cell line. The GO enrichment revealed that the DEGs were associated with biological processes related to fatty acids (**Figure** **5**A). In our pursuit of a more precise research target, we identified SREBF1, a pivotal gene in lipid synthesis, as a potential downstream target of PDGFC in the regulation of lipid metabolism and a contributor to PDAC carcinogenesis (Figure 5B; Figure S5A,B, Supporting Information). The mRNA level of SREBF1 also substantially decreased following PDGFC knockdown in another PDAC cell line SW1990 (Figure 5C).

Existing studies have underscored the indispensability of PDGFR for the biological functions of the PDGF family.^[^ ^17^ ^]^ Therefore, we performed KEGG and GSEA enrichment among downregulated DEGs. The results suggested that the PI3K/AKT signaling pathway could be the downstream regulatory pathway of PDGFC, with a concurrent downregulation in the fatty acid metabolism pathway (Figure 5D,E). To further verify the above results, we conducted a series of experiments. Remarkably, we observed that PDGFC activated the PI3K/AKT signaling pathway by binding to and phosphorylating its receptor PDGFR. In detail, the PDGFR/PI3K/AKT pathway was down‐regulated after PDGFC silencing and the expression level of precursor and mature form of SREBP1 (pSREBP1 and mSREBP1) were also decreased (Figure 5F). In addition, we investigated the relationship between PDGFC and SREBF1 expression. The results indicated a positive correlation in the expression of PDGFC and SREBF1 mRNA in PDAC (Figure S5C, Supporting Information). Moreover, the opposite phenomenon was observed after processing with recombinant human PDGF‐CC protein (Figure S5D, Supporting Information). To further elucidate the pivotal role of PI3K/AKT signaling pathway in PDGFC regulation of SREBP1, we treated PDAC cells with capivasertib, a specific AKT inhibitor (TargetMol, Shanghai, China). The results suggested that AKT activation was significantly inhibited after capivasertib treatment (Figure S5E, Supporting Information), and the expression levels of pSREBP1 and mSREBP1 were down‐regulated synchronously (Figure 5G,H). Furthermore, we treated PDAC cells with capivasertib on the basis of overexpression of PDGFC. We found that inhibition of AKT activation by capivasertib effectively rescued the up‐regulation of SREBP1 protein induced by overexpression of PDGFC (Figure 5I). Finally, we assessed the levels of phosphorylated‐AKT (p‐AKT) and SREBP1 proteins in xenograft tumors using IHC staining. The results demonstrated a simultaneous decrease in the protein levels of p‐AKT and SREBP1 in PDGFC knockdown xenograft tumors compared to the control tumors (Figure 5J,K). These experiments confirmed that SREBP1 is regulated by PDGFC via PI3K/AKT pathway in PDAC.

To clarify that PDGFC promoted PDAC lipid biosynthesis by regulating SREBP1, we performed Nile red staining. The results revealed that SREBF1 overexpression rescued the fluorescence intensity of lipid droplets under the conditions of PDGFC knockdown (Figure S5F, Supporting Information). Notably, the intracellular triglyceride level was also restored by SREBF1 overexpression (Figure S5G, Supporting Information). Our data strongly suggests that PDGFC regulates fatty acid metabolism through the PI3K/AKT/SREBP1 signaling pathway.

### 2.6 PDGFC Facilitates PDAC Progression by Upregulating SREBP1 Expression

**Figure 6:**
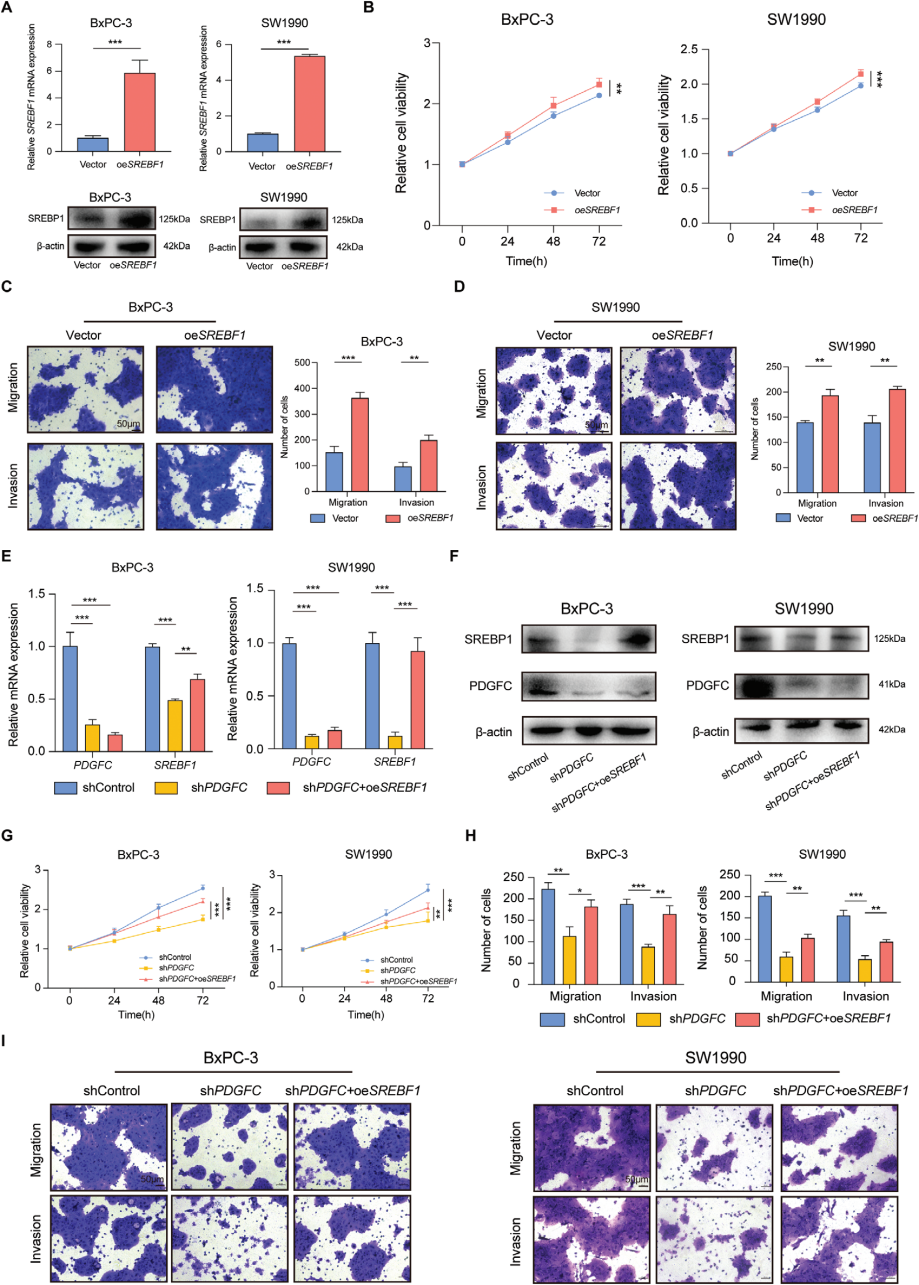
PDGFC facilitates PDAC progression by upregulating SREBP1 expression. A) The mRNA and protein level after SREBF1 overexpression in BxPC‐3 and SW1990 cells. B) Cell growth curve after SREBF1 overexpression in PDAC cells. C) Cell migration and invasion ability after SREBF1 overexpression in BxPC‐3 cells. D) Cell migration and invasion ability after SREBF1 overexpression in SW1990 cells. E) The mRNA level after PDGFC silencing and PDGFC silencing with SREBF1 overexpression confirmed by RT‐qPCR. F) The protein level after PDGFC silencing and PDGFC silencing with SREBF1 overexpression confirmed by western blotting. G) Cell growth curve after PDGFC silencing and PDGFC silencing with SREBF1 overexpression. H) Statistical analysis of cell migration and invasion ability. I) Representative images of cell migration and invasion ability in BxPC‐3 and SW1990 cells. Data are presented as mean ± SD (n = 3). ^*^ *p* < 0.05; ^**^ *p* < 0.01; ^***^ *p* < 0.001 according to Student's *t*‐test.

To validate the role of SREBP1 in PDAC malignant progression, we overexpressed SREBF1 in PDAC cell lines, and the efficiency was confirmed by RT‐qPCR and western blotting (**Figure** **6**A). Following SREBF1 overexpression, we observed a mild increase in cell growth rate, migration, and invasion ability in both BxPC‐3 and SW1990 cells (Figure 6B–D). These findings suggest that SREBF1 functions as an oncogenic factor in PDAC, promoting its metastatic potential.

Subsequently, SREBF1 was overexpressed in PDGFC stable knockdown PDAC cells using a overexpression plasmid. The mRNA and protein levels of SREBF1 before and after overexpression were confirmed by RT‐qPCR and western blotting (Figure 6E,F). As anticipated, the overexpression of SREBF1 rescued the proliferation of PDGFC knockdown PDAC cells (Figure 6G). Simultaneously, overexpression of SREBF1 partially restored the invasion and migration of PDGFC knockdown PDAC cells (Figure 6H,I). These findings support the notion that PDGFC promotes PDAC progression by upregulating the expression of SREBP1.

### 2.7 The Lipid Metabolism Inhibitor Betulin Effectively Mitigates the Metastatic Process of PDAC

**Figure 7:**
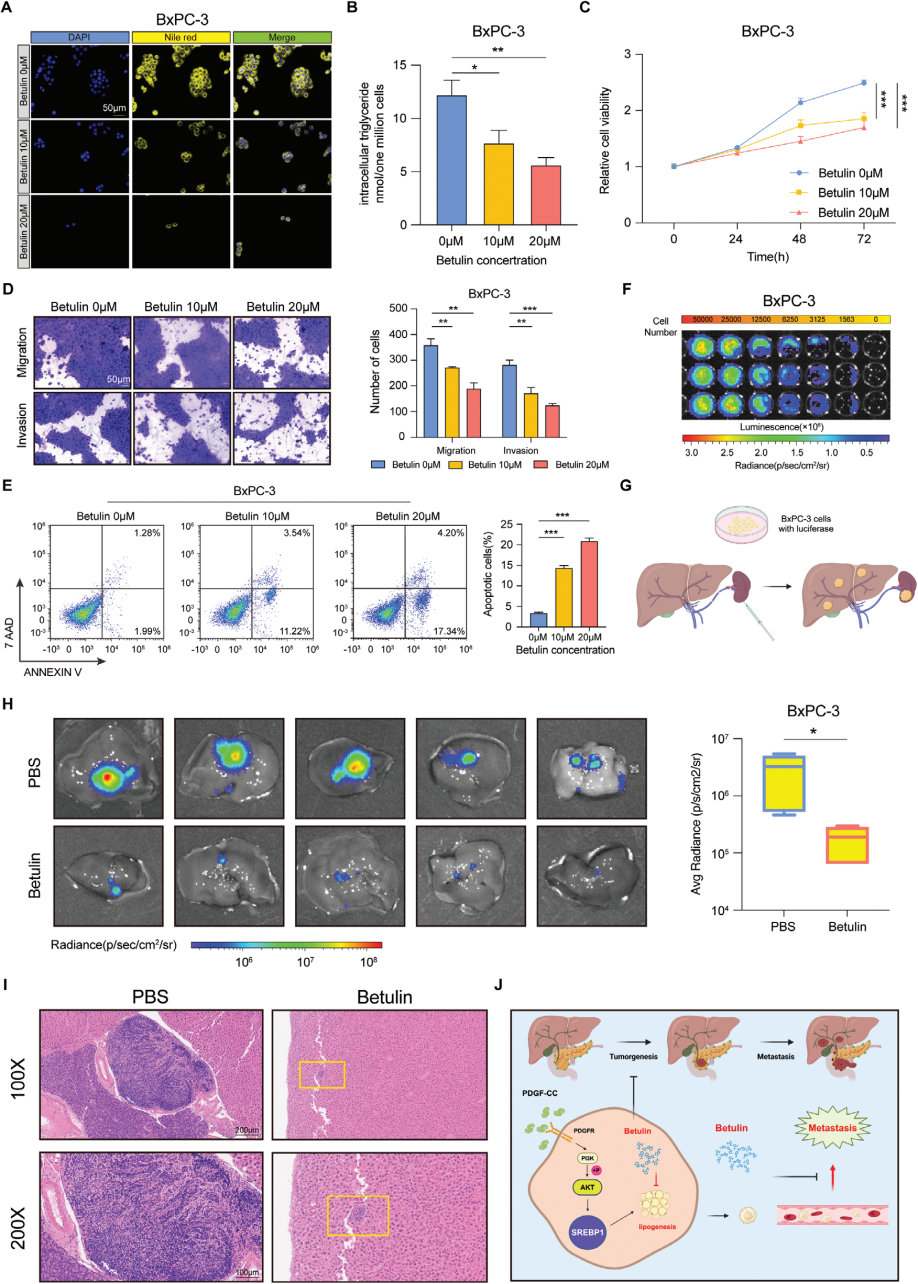
The lipid metabolic inhibitor betulin effectively mitigates the metastatic process of PDAC. A) Representative images of Nile red staining in BxPC‐3 cells treated with indicated dose of betulin. B) Intracellular triglyceride level in BxPC‐3 cells treated with indicated dose of betulin. C) Cell growth curve of BxPC‐3 cells treated with indicated dose of betulin. D) Cell migration and invasion ability of BxPC‐3 cells treated with indicated dose of betulin. E) Cell apoptotic rate of BxPC‐3 cells treated with indicated dose of betulin. F) The fluorescent intensity with different number of BxPC‐3 cells. G) The diagram of construction of PDAC liver metastasis model created with BioRender.com. H) Bioluminescent images of livers and statistical analysis of fluorescent intensity from different treatment group. I) Representative liver images of HE staining from different group. J) Schematic illustration of the proposed model, showing PDGFC upregulates SREBP1 induced lipid synthesis via PI3K/AKT signaling pathway, thereby promoting proliferation and metastasis of PDAC. The graphic was created with BioRender.com. Data are presented as mean ± SD (n = 3 in B–E and n = 5 in H). ^*^ *p* < 0.05; ^**^ *p* < 0.01; ^***^ *p* < 0.001 according to Student's *t*‐test.

Betulin is an inhibitor of SREBP1. To validate the inhibitory function of betulin (TargetMol, Shanghai, China) on PDAC lipid synthesis, PDAC cells were stained with Nile red, revealing a reduction in lipid droplets as the betulin concentration increased (**Figure** **7**A; Figure S6A, Supporting Information). The intracellular triglyceride level was also decreased after betulin treatment (Figure 7B; Figure S6B, Supporting Information). Subsequently, PDAC cells were treated with varying concentrations of betulin. The results indicated that betulin inhibited the proliferation, migration, and invasion of PDAC cells (Figure 7C,D; Figure S6C,D, Supporting Information). The apoptotic rate was also significantly increased after betulin treatment (Figure 7E; Figure S6E, Supporting Information). Additionally, an orthotopic xenograft model experiment in nude mice was conducted to corroborate the effects of the lipid metabolic inhibitor betulin in PDAC in vivo. PDAC cells with luciferase plasmids were successfully constructed (Figure 7F) and injected into the spleen to establish a liver metastatic tumor model of PDAC in nude mice (Figure 7G). Following treatment with betulin or PBS, the mice were sacrificed, and liver bioluminescent signals were assessed. The results revealed reduced bioluminescent signals after betulin treatment, signifying the crucial role of betulin in inhibiting PDAC liver metastasis (Figure 7H). Moreover, HE staining demonstrated the impactful effect of betulin on inhibiting PDAC metastasis from a microscopic perspective (Figure 7I), with no significant impact on the hearts, lungs, and kidneys at this dosage (Figure S7, Supporting Information). Collectively, the in vitro and in vivo experiments highlight the pivotal role of the lipid metabolic inhibitor betulin in mitigating PDAC proliferation and metastasis, suggesting that PDGFC‐induced lipid metabolism represents a promising therapeutic target for PDAC therapy.

## 3 Discussion

Growth factors are known to play a crucial role in the regulation of cell growth and proliferation, and their dysregulation has been implicated in cancer development and metastasis. Understanding the complex mechanisms behind tumor progression and metastasis may provide potential targets for cancer therapy. We found that PDGFC is a key molecular in the malignant progression of PDAC and it is an upstream factor of lipid biosynthesis. Previous study has already illustrated that PDGFC, like other PDGFs, performs their biological functions by activating their receptor PDGFR, which is located on the cell membrane. After PDGF activates PDGFR, the receptor phosphorylates the downstream PI3K/AKT or RAS/MAPK signaling pathway and exerts its biological functions.^[^ ^18^ ^]^ Abnormal activation of PI3K/AKT signaling pathway or MAPK pathway both are associated with poor prognosis and chemoresistance in malignant tumors.^[^ ^19^ ^]^ While it is currently understood that PDGFC exerts its biological functions by activating downstream pathways via binding to PDGFR, the specific mechanisms by which PDGFC promotes tumor proliferation and metastasis are still under investigation. Our study has uncovered that PDGFC can induce fatty acid biosynthesis in PDAC by activating its classical pathway, thereby facilitating the malignant behavior of PDAC.

Studies have indicated a potential association between PDGFC and the development of diverse tumors. In their research, Hyunho et al. discovered that PDGFC activates PDGFR and its downstream cascade in a paracrine manner, thereby promoting the proliferation and metastasis of gastrointestinal stromal tumors (GIST). Additionally, the combined use of PI3K/mTOR inhibitors and imatinib demonstrated a synergistic inhibition of the malignant behavior of GIST.^[^ ^20^ ^]^ In estrogen receptor (ER)‐positive breast cancer, Turrell et al. discovered that low‐level PDGFC expression in young mice maintained the dormancy of disseminated tumor cells. However, in an environment with high PDGFC levels, such as in old or fibrotic lungs, it could trigger the proliferation of tumor cells. This effect might be alleviated by suppressing PDGFRa or blocking PDGFC.^[^ ^21^ ^]^ In non‐small cell lung cancer, PDGFC or its receptor PDGFRa played a pivotal role in promoting tumor proliferation. Blocking this signaling pathway was found to be an effective strategy for inhibiting tumor growth.^[^ ^22^ ^]^ These studies suggest that PDGFC may be implicated in the malignant progression of various tumors; however, the specific mechanisms of tumorigenesis vary greatly among them. In our study, we observed abnormal high expression of PDGFC in PDAC, and this elevated expression was positively correlated with a poor prognosis. PDGFC knockdown was found to significantly inhibit the proliferation and metastasis of PDAC both in vitro and in vivo. Mechanistically, PDGFC promotes fatty acid synthesis by activating the PI3K/AKT signaling pathway, thereby playing a crucial role in tumorigenesis and metastasis. Inhibiting the lipid metabolism reprogramming could impair the proliferation and metastasis of PDAC.

A well‐known characteristic that distinguishes malignant tumors from their parental cells is metabolic reprogramming, which involves an enhanced capacity to synthesize major macromolecules necessary for generating new cells, such as proteins, nucleotides, and lipids.^[^ ^23^ ^]^ Cancer cells sustain their accelerated proliferation, migration, and invasion within tumor microenvironments, where the nutrient supply constantly fluctuates due to tumor development. Cancer cells can adapt to this dynamic environment by undergoing alterations in lipid metabolism. Intracellular lipids mainly include fatty acids and cholesterol, with fatty acid metabolism being a crucial component of lipid metabolism. Fatty acid metabolism involves two processes: the conversion of lipids into energy and the provision of primary metabolites, as well as the synthesis of essential molecules. Moreover, fatty acid metabolism is essential for the formation of cell membranes, the storage of energy within the organism, and the transmission of signals.^[^ ^24^ ^]^ This metabolic reprogramming supports cancer cells survival under nutrient‐poor conditions encountered during metastasis.^[^ ^25^ ^]^ Therefore, targeting lipid metabolism may play a crucial role in the treatment of malignant tumors.

The protein SREBP1, encoded by SREBF1, is a member of the SREBP family. SREBPs regulate the expression of factors involved in the uptake and synthesis of cholesterol, fatty acids, and phospholipids.^[^ ^26^ ^]^ Dysregulation of SREBPs is frequently associated with hyperactive fatty‐acid synthesis and lipogenesis pathways in cancers.^[^ ^24^ ^]^ In this family, SREBP1 primarily stimulates the synthesis of fatty acid, while SREBP2 predominantly acts on cholesterol biosynthesis genes.^[^ ^27^ ^]^ SREBPs can bind to the promoter region of key genes involved in lipid metabolism, thereby increasing lipid biosynthesis. Previous studies have demonstrated that SREBPs can be activated by the insulin activated PI3K/AKT pathway, resulting in biosynthesis of cholesterol and fatty acids.^[^ ^26^, ^28^ ^]^ Furthermore, Ricoult et al. reported that the PI3K/AKT signaling pathway could serve as the upstream regulatory mechanism of SREBP1.^[^ ^29^ ^]^ In our study, KEGG and GO enrichment analysis through RNA‐seq showed that PDGFC could regulate the fatty acid metabolism process. Subsequent analysis indicated that PDGFC might influence fatty acid accumulation and oxidation by modulating the PDGFR‐activated PI3K/AKT signaling pathway, representing a novel upstream ligand for SREBP1 regulation. In detail, PDGFC could promote the transcription of SREBF1 through PI3K/AKT signaling pathway and then promote the maturation of SREBP1 protein in PDAC, which is consistent with previous studies.^[^ ^28^, ^30^ ^]^ The findings shed light on the mechanism underlying PDGFC‐induced lipid metabolism reprogramming in PDAC.

Metastasis is responsible for more than 90% of cancer‐related deaths.^[^ ^31^ ^]^ PDAC is among the most lethal cancers, largely attributed to its high incidence of metastasis. Obesity stands out as one of the few recognized risk factors for PDAC, correlating with an unfavorable prognosis.^[^ ^32^ ^]^ Some studies have also discovered that high‐fat diets expedite carcinogenesis and intensify metastasis in mouse models of PDAC.^[^ ^33^ ^]^ As is widely recognized, the metastatic process is highly energy‐intensive. Therefore, cancer cells need to activate their fatty acid metabolic pathways to utilize these metabolites. Fatty acids are stored in the form of lipid droplets within cells. When the organism requires, lipases within the lipid droplets become activated, breaking down stored triglycerides into fatty acids and glycerol. Subsequently, energy is released through the process known as β‐oxidation of fatty acids. We observed a reduction in intracellular lipid droplets and a simultaneous decrease in the level of intracellular triglycerides following PDGFC knockdown. These results suggest that the reduction in PDGFC levels results in the down‐regulation of fatty acid metabolism, impacting intracellular energy metabolism and manifesting as a decrease or death of cells. Therefore, the metabolic changes that occur during tumor metastasis might provide a new therapeutic strategy to reduce tumor migration and invasion. This approach shows promise for enhancing outcomes in patients with metastatic cancer.

Nowadays, numerous studies have shown that natural compounds can serve as effective anti‐cancer treatments. Betulin, a natural compound derived from birch species, exhibits various pharmacologic effects, including anti‐inflammatory and anti‐osteoclastogenic effects.^[^ ^34^ ^]^ Furthermore, several studies have revealed that betulin also inhibits the proliferation and metastasis of cancer. In colorectal cancer, betulin inhibits liver metastasis by modulating the cell cycle, autophagy, and apoptosis.^[^ ^35^ ^]^ In advanced gastric cancer, betulin also inhibits tumor growth.^[^ ^36^ ^]^ According to recent research, betulin and its derivative, betulinic acid, possess various properties that can be utilized in the treatment of metabolic, infectious, cardiovascular, and neurological diseases.^[^ ^37^ ^]^ In the context of metabolic reprogramming, betulin reduces lipid accumulation by inhibiting the activity of SREBP1, the key enzyme involved in lipid biosynthesis.^[^ ^38^ ^]^ To illustrate the anti‐tumor effect of betulin in PDAC, we treated the PDAC cell lines with different doses of betulin. Interestingly, betulin inhibited the proliferation and metastasis of PDAC in vitro. To confirm the effect of betulin on inhibiting tumor metastasis in vivo, we established a liver metastasis model in nude mice. Remarkably, we observed that betulin not only exhibited a potent anti‐tumor effect in vitro but also demonstrated the ability to inhibit tumor metastasis in vivo. This metastasis‐inhibiting effect is mediated by the suppression of lipid synthesis by betulin.

In conclusion, our study provides valuable insights into the regulatory role of PDGFC in lipid metabolism. PDGFC enhances fatty acid synthesis by upregulating SREBP1 activity, thereby activating the transcription of key enzymes of lipogenesis. The Lipid metabolic inhibitor, identified as a potential therapeutic agent capable of inhibiting tumor metastasis, holds promise for the development of more effective strategies for PDAC patients (Figure 7J). Further clinical studies are warranted to explore the clinical applicability of betulin when combined with first‐line chemotherapy drugs to enhance the prognosis of advanced PDAC patients and potentially address challenges posed by early metastasis in this devastating malignancy.

## 4 Experimental Section

### Patients’ Specimens

Paraffin‐embedded specimens from PDAC patients who underwent surgical resection were obtained from the First Affiliated Hospital of Sun Yat‐sen University (Guangzhou, China) for subsequent analysis of PDGFC expression. This study was approved by the IEC for Clinical Research and Animal Trials of the First Affiliated Hospital of Sun Yat‐sen University.

### Cell Lines and Cell Culture

The PDAC cell lines BxPC‐3 and SW1990 were purchased from Cellcook (Guangzhou, China). The two cell lines were cultured in RPMI 1640 (BasalMedia) media containing 10% fetal bovine serum (BasalMedia). Human embryonic kidney cells HEK293T were cultured in DMEM high glucose media (BasalMedia) containing 10% fetal bovine serum (BasalMedia) and used for subsequent construction of stable transfection cell lines with lentiviral vectors. The cells were maintained at 37 °C with 5% CO_2_ in a humid cell culture incubator.

### Cell Viability Assay

Cell viability was evaluated using the CellTiter‐Lumi II Luminescent Cell Viability Assay Kit (Beyotime, Shanghai, China). The PDAC cells with different treatments were seeded into a 96‐well plate at a density of 2000 cells per well. After adhesion, the baseline fluorescence value was measured according to the manufacturer's instructions. Fluorescence values were then measured at 24, 48, and 72 h. The fluorescence values were proportional to the number of cells and could represent the number of cells per well.

### Colony Formation Assay

The PDAC cells with different treatments were seeded into a six‐well plate at a density of 1000 cells per well and the medium was refreshed every 4 days. After ≈ 14 days’ culture, the medium was discarded, and the cells were washed twice with PBS before being fixed with 4% paraformaldehyde for 10 min. After washing twice with PBS, the cells were stained with 0.1% crystal violet solution for 30 min. The number of cell clones was photographed and counted.

### Apoptosis Assay

PDAC cells with different treatments were seeded into six‐well plate and cultured for 48 h. Then the cells were collected, washed, and stained by Annexin V‐APC/7‐AAD cell apoptosis assay kit (70‐AP105‐100, MultiSciences, Hangzhou, China) according to the manufacturer's instruction. After staining, the apoptosis rate was measured by flow cytometer (BD Bioscience).

### Migration and Invasion Assay

The PDAC cells with different treatments were seeded into a 24‐well plate containing transwell‐inserts at a density of 50000 cells per well. For invasion assay, the transwell inserts were coated with Matrigel (BD Biosciences, Bedford, MA, USA). Cells were cultured in culture medium without serum in the upper chamber, and medium containing 10% fetal bovine serum was used as a chemoattractant in the lower chamber. After 24–60 h, the medium was discarded, and the cells were washed twice with PBS solution before being fixed with 4% paraformaldehyde for 10 min. After washing twice with PBS, the cells were stained with 0.1% crystal violet solution for 30 min. Cells that did not migrate through the pores were removed by cotton swabs. The number of cells were photographed and counted.

### Immunohistochemistry (IHC) and Hematoxylin‐Eosin (HE) Staining

Tissues from PDAC patients and xenografted tumors from nude mice were fixed in 4% paraformaldehyde. The tissues were embedded, cut, and mounted on glass slides by Servicebio (Wuhan, China). IHC was carried out to detect the protein level of PDGFC (ab93899, Abcam, Shanghai, China), p‐AKT(28731‐1‐AP, proteintech, China), SREBP1 (66875‐1‐Ig, proteintech, China) and Ki67 (27309‐1‐AP, proteintech, China) in tissues as described previously.^[^ ^39^ ^]^ A DAB staining kit and HRP conjugated Goat Anti‐Rabbit or Anti‐mouse IgG (Servicebio, China) were used to detect the primary antibodies, and then the tissues were stained with hematoxylin (G1076‐500ML, Servicebio, China). The results were analyzed independently by two experienced observers. The final score was calculated as stain intensity (negative, 0; mild, 1; moderate, 2; severe, 3) multiplying stain area (negative, 0; ≤30%, 1; >30 and ≤60%, 2; >60%, 3). The dilution ratio of PDGFC, p‐AKT and SREBP1 antibodies was 1:200, the dilution ratio of Ki67 was 1:3000, and the dilution ratio of secondary antibody was 1:200. The HE staining was performed according to the manufacturer's instruction (G1076‐500ML, Servicebio, China).

### TUNEL Assay

TUNEL assay was performed using paraffin sections of tissues to assess apoptosis. First, the sections were soaked twice in xylene to fully dewaxing, followed by immersion in gradient ethanol for hydration. After cleaning with PBS, proteinase K solution of 20 µg mL^−1^ was incubated at 37 °C for 40 min and cleaned with PBS. Subsequently, the tissues were incubated with 0.3% Triton solution for 20 min at room temperature, followed by three times washes with PBS. The subsequent steps were followed by staining method provided by the manufacturer's protocol (Fluorescein Tunel Cell Apoptosis Detection Kit G1501, Servicebio, China). The results are represented by the number of TUNEL‐positive cells (green) in the field of view to represent the apoptosis of the tissues.

### Intracellular Triglyceride

The PDAC cells with different treatments were seeded into a six‐well plate. After two days culture, the cells were digested and counted. Then, intracellular triglyceride contents were analyzed by kits purchased from Applygen Technologies Inc. (E1013‐105, Beijing, China) according to the manufacturer's instructions. The intracellular triglyceride levels were normalized to the number of cells.

### Nile Red Stain

The PDAC cells with different treatments were seeded into a six‐well plate with a density of 10000 cells per well. After 48 h culture, the cells were washed twice by PBS and fixed in 4% paraformaldehyde solution. Then, the cells were stained with 5 µg ml^−1^ Nile red solution (TargetMol, Shanghai, China) for 8 min followed by PBS washing three times. Finally, the cells were stained by DAPI (Beyotime, Shanghai, China). The images were visualized by immunofluorescence microscopy (Leica DMI8).

### Enrichment Analysis

Kyoto Encyclopedia of Genes and Genomes (KEGG) Enrichment Analysis, Gene Set Enrichment Analysis (GSEA) and Gene Ontology (GO) Enrichment Analysis were employed to elucidate biological functions associated with PDGFC or PDAC malignant behavior. The KEGG analysis was performed by Sangerbox tools (http://vip.sangerbox.com/).^[^ ^40^ ^]^ The Go enrichment was conducted by the R software (version 4.2.1) with “DOSE”, “enrichplot”, “GOplot” and “clusterProfiler” package.^[^ ^41^ ^]^ For GESA enrichment, PDAC cells transfected with shControl plasmids were used as a control group and PDAC cells with PDGFC knocked‐down as an experimental group for comparison. Subsequently, the above data and the related pathway gene sets downloaded from the GSEA website (https://www.gsea‐msigdb.org/gsea) were imported into GSEA software (version 4.3.1 for macOS) for enrichment analysis and visualization.

### Statistical Analysis

GraphPad Prism (version 9.0 for macOS) and R software (version 4.2.1) were utilized for statistical analysis and visualization. The statistical data in this study were given as mean ± standard deviation (SD) and analyzed by Student's *t*‐test with statistical significance defined as *P* < 0.05. Sample size (n) for each statistical analysis was shown in the figure legend. The heatmap, volcano plot, and GO chord plot were analyzed and drawn online in BioLadder (bioladder.cn).^[^ ^42^ ^]^ The Venn plot was analyzed and drawn using the OmicShare tools (https://www.omicshare.com/tools). The Kaplan‐Meier curve was drawn by the “survminer” and “survival” packages in R software. Spearman's correlation coefficient was used to assess the correlations between two groups.

Additional information on methods is available in the supporting information file.

### Ethics Approval Statement

The clinical samples used in the study were collected from the First Affiliated Hospital of Sun Yat‐sen University and the experiments were approved by the IEC for Clinical Research and Animal Trials of the First Affiliated Hospital of Sun Yat‐sen University ([2023]841). All animal experiments were approved by the IEC for Clinical Research and Animal Trials of the First Affiliated Hospital of Sun Yat‐sen University ([2023]121).

## Conflict of Interest

The authors declare no conflict of interest.

## Author Contributions

Y.‐H.S., Z.‐D.L., M.‐J.M., G.‐Y.Z. contributed equally to this work. X.‐Y.Y. conceived and designed the study. Y.‐H.S., Z.‐D.L., M.‐J.M., G.‐Y.Z. performed experiments and analyzed the results. Y.‐H.S., Y.‐Q.Z., J.‐Q.W., Y.‐Y.‐H.Y., X.‐T.H., J.‐Y.Y., F.‐X.L., and X.‐Y.W. assisted in the collection and analysis of clinical samples. X.‐Y.Y. and Q.‐C.X. supervised and guaranteed the study. Y.‐H.S., Q.‐C.X., and X.‐Y.Y. prepared and reviewed the manuscript. All authors approved the final version of the manuscript.

## Supporting information

Supporting Information

## Data Availability

The data that support the findings of this study are available from the corresponding author upon reasonable request.
