# Supplementary material for: Platelet‐Derived Growth Factor C Facilitates Malignant Behavior of Pancreatic Ductal Adenocarcinoma by Regulating SREBP1 Mediated Lipid Metabolism
Source: Adv Sci (Weinh). 2024 Sep 3;11(40):2407069. doi: 10.1002/advs.202407069 (PMC11516052; doi:10.1002/advs.202407069)
Supplement: Supplementary file 1 — Supporting Information [file ADVS-11-2407069-s001.pdf]

ADVANCED SCIENCE Open Access

Supporting Information

for *Adv. Sci.*, DOI 10.1002/advs.202407069

Platelet-Derived Growth Factor C Facilitates Malignant Behavior of Pancreatic Ductal Adenocarcinoma by Regulating SREBP1 Mediated Lipid Metabolism

Yin-Hao Shi, Zhi-De Liu, Ming-Jian Ma, Guang-Yin Zhao, Ying-Qin Zhu, Jie-Qin Wang, Yang-Yin-Hui Yu, Xi-Tai Huang, Jing-Yuan Ye, Fu-Xi Li, Xi-Yu Wang, Qiong-Cong Xu and Xiao-Yu Yin**

Supplementary information for

Platelet-derived Growth Factor C Facilitates Malignant Behavior of Pancreatic Ductal Adenocarcinoma by Regulating SREBP1 Mediated Lipid Metabolism

Yin-Hao Shi, Zhi-De Liu, Ming-Jian Ma, Guang-Yin Zhao, Ying-Qin Zhu, Jie-Qin Wang, Yang-Yin-Hui Yu, Xi-Tai Huang, Jing-Yuan Ye, Fu-Xi Li, Xi-Yu Wang, Qiong-Cong Xu*, Xiao-Yu Yin*

This file contains following information:

Supplemental Methods

Figures S1-7

Table S1

Supplemental Methods

Stable transfection: The shRNA (short hairpin RNA) of PDGFC was synthesized from Sangon Biotech (Shanghai), with the following sequences: shPDGFC #1: CCTCATACTTATCCAAGAAAT; shPDGFC #2: CCTCAGCATGAGAGAATTATT. The overexpression SREBF1 plasmid was purchased from MiaoLingPlasmid (Wuhan, China). The overexpression PDGFC plasmid was purchased from Hanbio (Shanghai, China). The stable transfection was carried out as follows: according to the manufacturer's instruction, the plasmid of PDGFC shRNA or the overexpressing SREBF1 plasmid along with its corresponding negative control plasmid were transfected into cells by lentivirus using the transfection reagent PEI (Servicebio, Wuhan, China). After 24 hours of transfection, the cells were refreshed with fresh medium and seeded into 60 mm culture dishes. For stably knockdown and overexpression cell lines, the cells were selected with puromycin (Biosharp, Hefei, China) or G418 (TargetMol, Shanghai, China) for two generations, respectively.

RNA sequencing (RNA-seq): The RNA of the target sample was extracted by VeZol Reagent (Vazyme, Nanjing, China). The RNA quality was checked by Agilent 2200 and kept at −80°C. The RNA with RIN (RNA integrity number) > 7.0 is acceptable for cDNA library construction. The cDNA libraries were constructed for each RNA sample using the VAHTS Universal V6 RNA-seq Library Prep Kit for Illumina (Vazyme, Nanjing, China) according to the manufacturer's instructions. Generally, the protocol consists of the following steps: Poly-A containing mRNA was purified from 1μg total RNA using oligo (dT) magnetic beads and fragmented into 200-600 bp using divalent cations at 85℃ for 6 min. The cleaved RNA fragments were used for first- and second-strand complementary DNA (cDNA) synthesis. dUTP mix was used for second-strand cDNA synthesis, which allows for the removal of the second strand. The cDNA fragments were end repaired, A-tailed and ligated with indexed adapters. The ligated cDNA products were purified and treated with uracil DNA glycosylase to remove the second-strand cDNA. Purified first-strand cDNA was enriched by PCR to create the cDNA libraries. The libraries were quality controlled with Agilent 2200 and sequenced

by DNBSEQ-T7 on a 150 bp paired-end run (NovelBio, Shanghai, China). DESeq2 algorithm was used to quantify transcription levels and identify differentially expressed genes using a cut-off of $P < 0.05$.

RT-qPCR: The total RNA of indicated cells with different treatment was extracted using VeZol Reagent (Vazyme, Nanjing, China). RNA reverse transcription was conducted with HiScript II Q RT SuperMix for qPCR (Vazyme, Nanjing, China) according to the manufacturer's instructions. The ChamQ Universal SYBR qPCR Master Mix（Vazyme, Nanjing, China）was used for RT-qPCR analysis. The relative expression levels were detected by QuantStudio 6 Flex Real-Time PCR Systems (Applied Biosystems, USA) and analyzed according to $2^{-\Delta\Delta CT}$. The Primers were listed in the Table S1.

Western blotting: Total proteins were extracted from cells with different treatment by Cell lysis buffer for Western and IP (Beyotime, Shanghai, China) containing protease inhibitors (TargetMol, Shanghai, China) and phosphatase inhibitors (CWBIO, Beijing, China) according to manufacturers' instructions. Protein was quantified using the BCA Protein Assay Kit (Thermo Scientific, USA) and boiled for 10 min at 95 °C. Protein samples were separated using FuturePAGETM precast gels (ACE Biotechnology, Nanjing, China), then transferred to polyvinylidene fluoride (PVDF) membrane blocked by 5% skim milk in Tris-buffered saline containing Tween-20 (TBST) solution at room temperature for 2 hours. Membranes were incubated with primary antibodies at 4 °C overnight with gently rocking, followed by incubation with horseradish peroxidase (HRP)-conjugated secondary antibodies (Servicebio, Wuhan, China) for 1 hour at room temperature before visualization by Enhanced Chemiluminescent kit (New Cell & Molecular Biotech, Suzhou, China). The anti-PDGFC antibody (ab93899) was purchased from abcam (Abcam, China), the anti-β-actin antibody (66009-1-Ig), anti-p-AKT antibody (28731-1-AP) and anti-SREBF1 antibody (66875-1-Ig) were obtained from proteintech (Wuhan, China). The anti-p-PDGFRa antibody (AP0568-50) and anti-PDGFRa (A2103-50) were purchased from ABclonal Technology. Anti-AKT antibody (#4691) were purchased from Cell Signaling Technology. β-actin was used as a loading control.

Animal experiment: The PDAC cell lines (5×10^6) with different treatment were

suspended by 100 μL PBS and injected into the right flank of the 4-6 weeks old female BALB/c nude mice (Animal Experiment Center of the First Affiliated Hospital of Sun Yat-sen University) subcutaneously. After approximately 7 days, the tumor volume was measured every 4 days by a caliper and calculated as (length × width^2)/2. About three weeks later, the mice were sacrificed, and xenograft tumors were then excised, fixed, weighed, photographed, and stored.

For the construction of liver metastasis model, female BALB/c nude mice were anesthetized by pentobarbital (50 mg/kg i.p.) and the surgical field was sterilized. The mice were placed in the right decubitus position, and a 0.5-1cm surgical incision was made below the left costal margin to access the abdominal cavity. Carefully apply pressure to push the spleen out of the abdominal cavity. Next, approximately 1x10^6 PDAC cells with luciferase activity suspended in 50μl/mouse PBS were injected into the lower pole of the spleen. Press the injection site with a sterilized cotton ball for 5 minutes after injection. Finally, suture the peritoneum and skin in layers. Penicillin/streptomycin solution (Gibco, Shanghai, China) was applied to the wound and the wound was sterilized with iodophor. The metastasis was observed by multimodal imaging system for living animals (AniView100, Guangzhou, China). D-Luciferin was bought from Goldbio (115144-35-9). Betulin (10mg/kg) was orally administrated to the nude mice every day for 14 days. All animal experiments were carried out with the approval of the IEC for Clinical Research and Animal Trials of the First Affiliated Hospital of Sun Yat-sen University.

Supplementary Figures

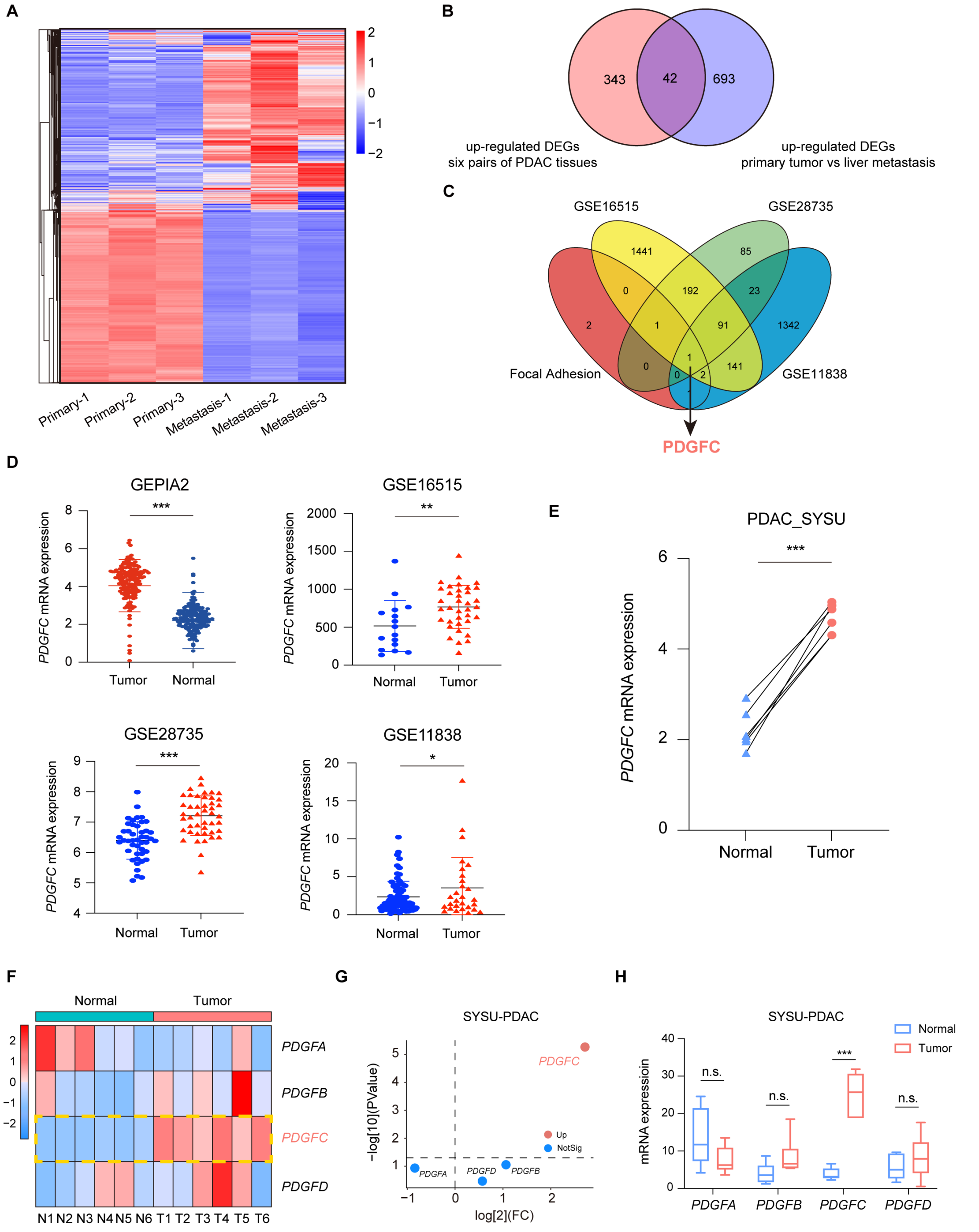

Figure S1. PDGFC is a potential predictor of metastasis in PDAC.

A. Heatmap of the DEGs in RNA sequencing results of primary tumor tissues and liver metastatic tumor tissues.

B. Venn diagram of the intersection of the up-regulated DEGs between PDAC tumor tissues and liver metastatic tumor tissues.

C. Venn diagram of the intersection of focal adhesion related genes and up-regulated

DEGs in three GEO datasets.

D. The mRNA expression of PDGFC in public datasets.

E. The mRNA expression of PDGFC in PDAC is higher than that in normal pancreatic tissue.

F. Heatmap showing the expression changes of PDGF family members between six pairs of pancreatic tumor and normal tissues.

G. Volcano plot showing the fold-change and p values of PDGF family members between six pairs of pancreatic tumor tissues and normal tissues.

H. Box plot showing the expression changes of PDGF family members between six pairs of pancreatic tumor and normal tissues. Data are presented as mean ± SD. * $p<0.05$; ** $p<0.01$; ***$p<0.001$ according to Student's t test. n.s.: no significance.

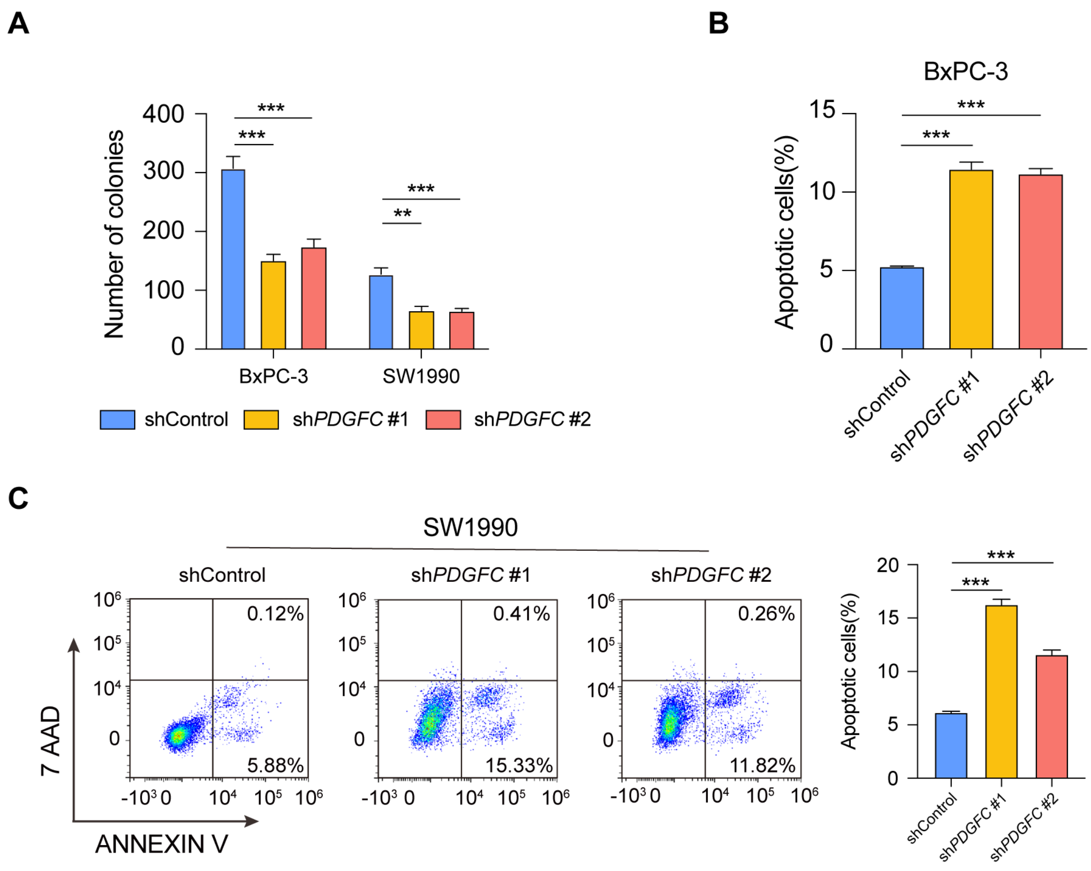

Figure S2. PDGFC knockdown inhibits PDAC progression *in vitro*.

A. Statistical analysis of colony-forming assays after PDGFC silencing in BxPC-3 and SW1990 cells.

B. Statistical analysis of apoptotic assays of BxPC-3 after transfected with shPDGFC or shControl.

C. Apoptotic assays of SW1990 cells after transfected with shPDGFC or shControl. Data are presented as mean ± SD (n = 3). ** $p<0.01$; ***$p<0.001$ according to Student's t test.

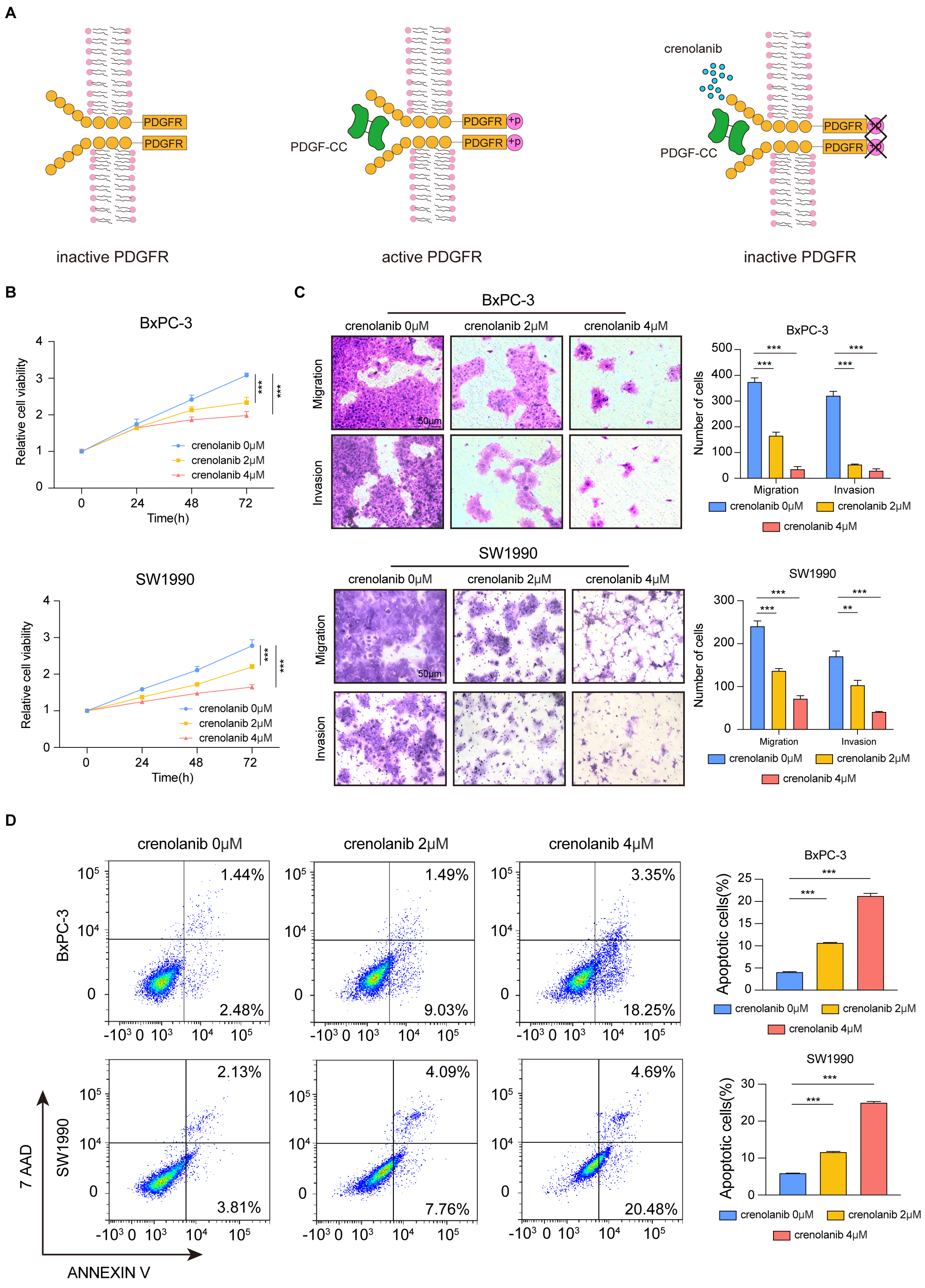

Figure S3. Crenolanib inhibits the malignant behavior of PDAC cells.

A. Schematic illustration of crenolanib suppression of PDGFC signal.

B. Cell growth curve of PDAC cells treated with the indicated dose of crenolanib.

C. Cell migration and invasion ability of PDAC cells treated with the indicated dose of crenolanib.

D. Cell apoptotic rates of PDAC cells treated with the indicated dose of crenolanib. Data are presented as mean ± SD (n = 3). ** $p<0.01$; ***$p<0.001$ according to Student's t test.

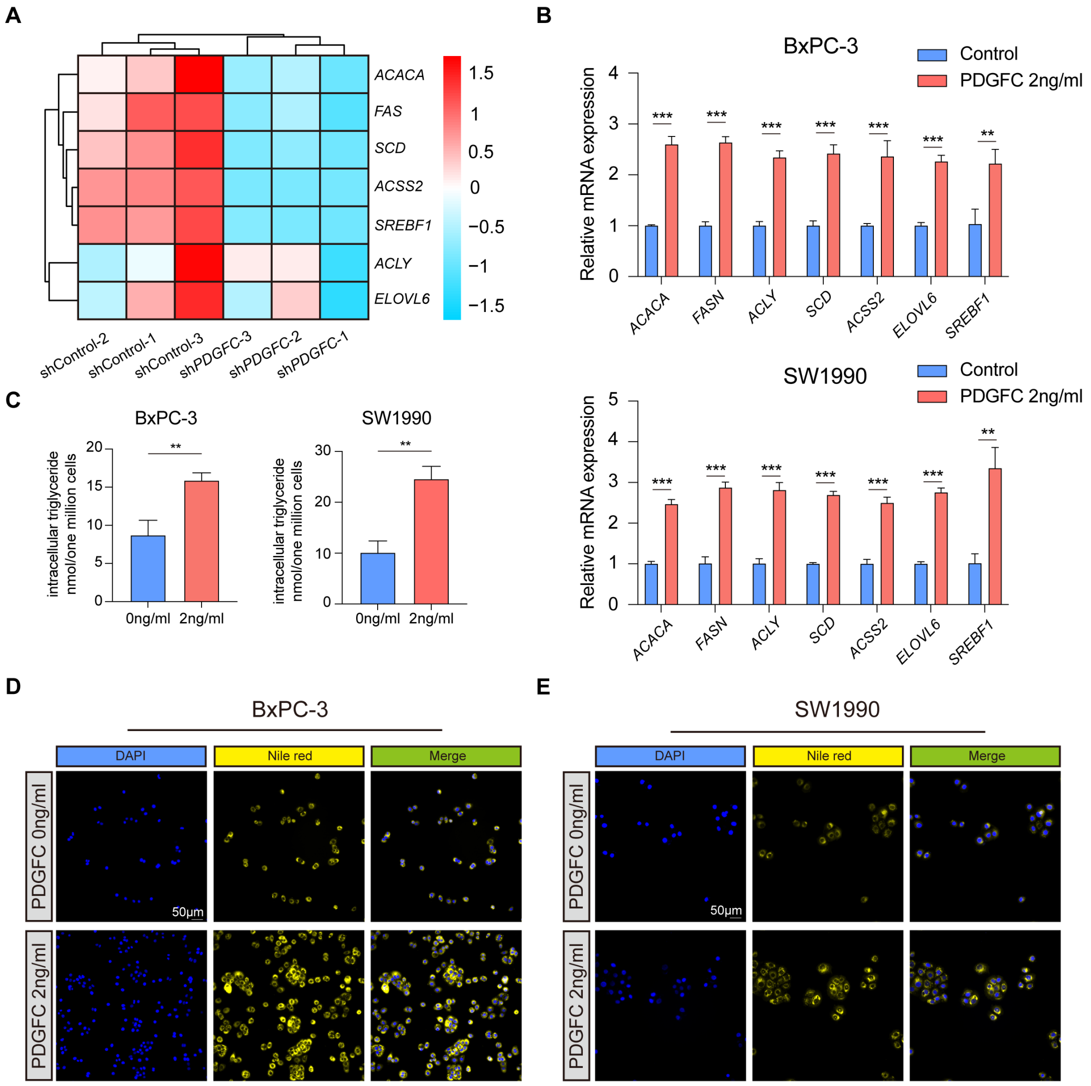

Figure S4. Fatty acid metabolism is a downstream crucial target of PDGFC in PDAC.

A. Heatmap of the lipid synthesis-related genes in RNA sequencing results of PDGFC silencing or not in BxPC-3 cells.

B. The mRNA levels of lipid synthesis-related key genes after treated with recombinant human PDGF-CC protein.

C. The intracellular triglyceride level in BxPC-3 and SW1990 cells treated with recombinant human PDGF-CC protein.

D. Representative images of Nile red stain in BxPC-3 cells treated with recombinant human PDGF-CC protein.

E. Representative images of Nile red stain in SW1990 cells treated with recombinant human PDGF-CC protein. Data are presented as mean ± SD (n = 3 in B and C). **

p<0.01; ***p<0.001 according to Student's t test.

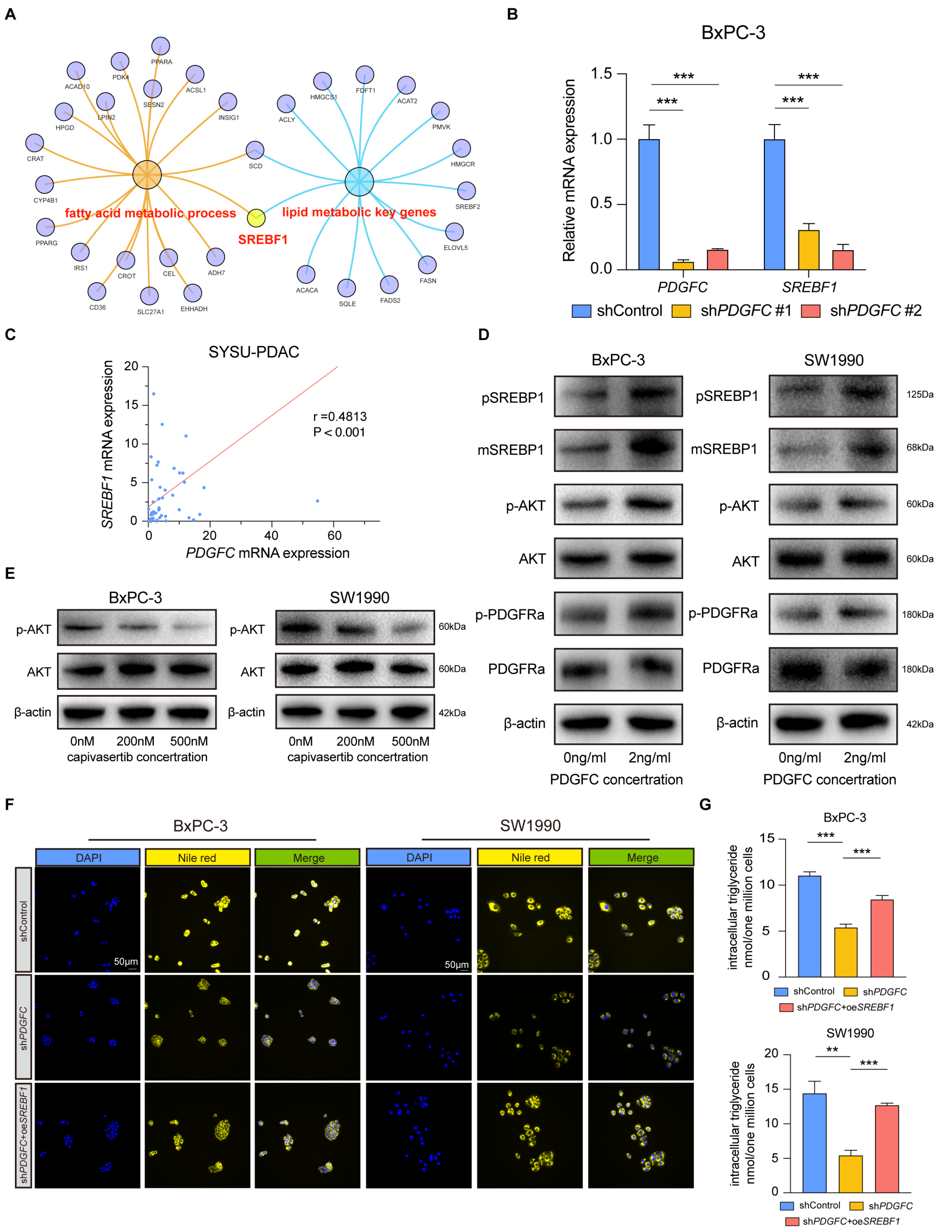

Figure S5. PDGFC regulates SREBP1 via PI3K/AKT signaling pathway to promote lipid biosynthesis.

A. Venn diagrams show the lipid metabolism key genes after PDGFC silencing in BxPC-3 cells analyzed by OmicShare tools (https://www.omicshare.com/tools).

B. The mRNA level of SREBF1 after PDGFC silencing in BxPC-3 cells confirmed by RT-qPCR.

C. The positive correlation of PDGFC mRNA and SREBF1 mRNA in PDAC according to spearman's correlation coefficient.

D. The protein level of PI3K/AKT signaling pathway and SREBP1 were confirmed by western blotting after human recombinant PDGFC protein treatment.

E. The protein level of p-AKT and AKT were confirmed by western blotting after capivasertib treatment.

F. Representative images of Nile red staining in PDAC cells with different treatment.

G. Intracellular triglyceride level in PDAC cells with different treatment. Data are presented as mean ± SD (n = 3 in B and G). ** $p<0.01$; ***$p<0.001$ according to Student's t test.

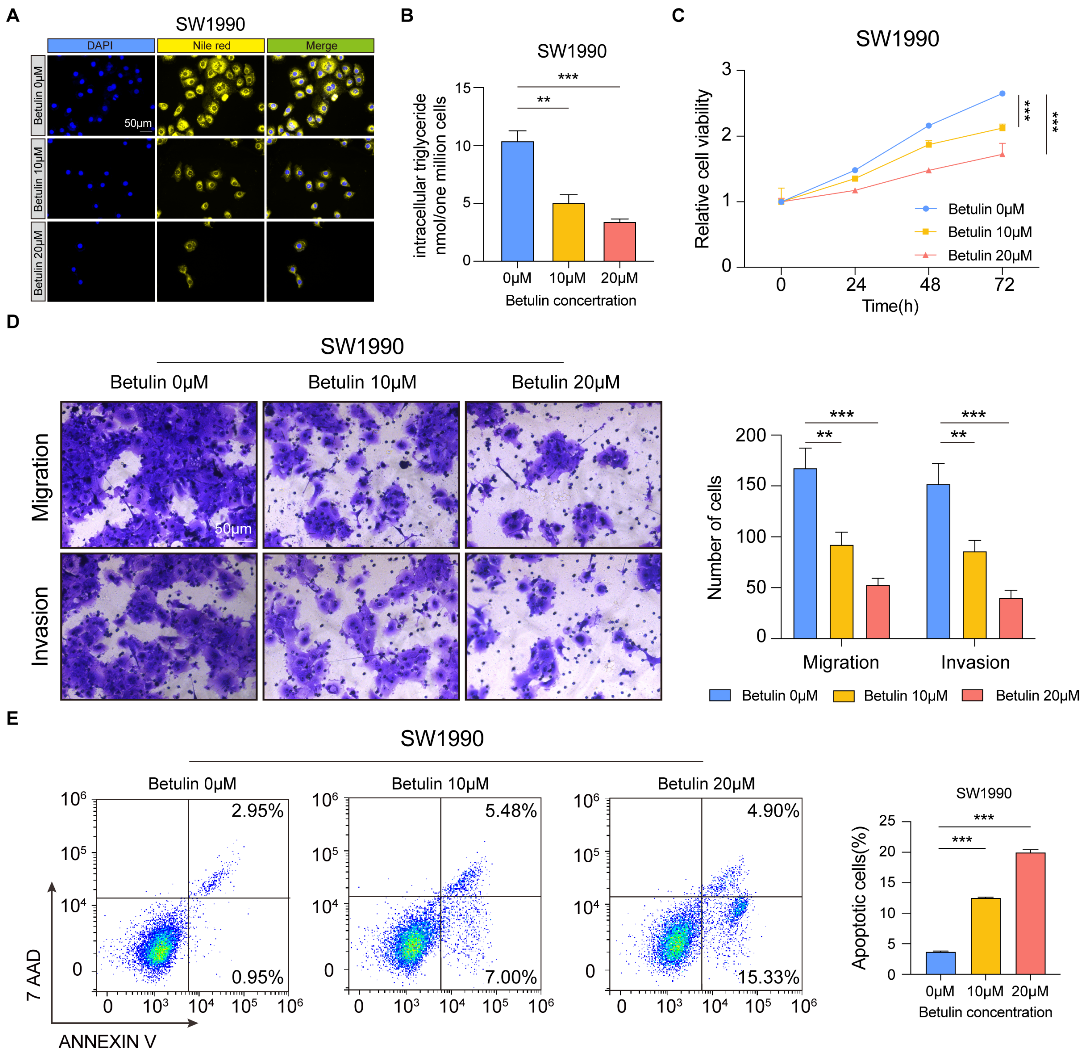

Figure S6. The lipid metabolic inhibitor betulin effectively mitigates the metastatic process of PDAC.

A. Representative images of Nile red staining in SW1990 cells with indicated dose of betulin treatment.

B. Intracellular triglyceride level in SW1990 cells with indicated dose of betulin treatment.

C. Cell growth curve of SW1990 cells treated with indicated dose of betulin.

D. Cell migration and invasion ability of SW1990 cells treated with indicated dose of betulin.

E. Cell apoptotic rates of SW1990 cells treated with indicated dose of betulin. Data are presented as mean ± SD (n = 3 in B to E). ** p<0.01; ***p<0.001 according to Student's t test.

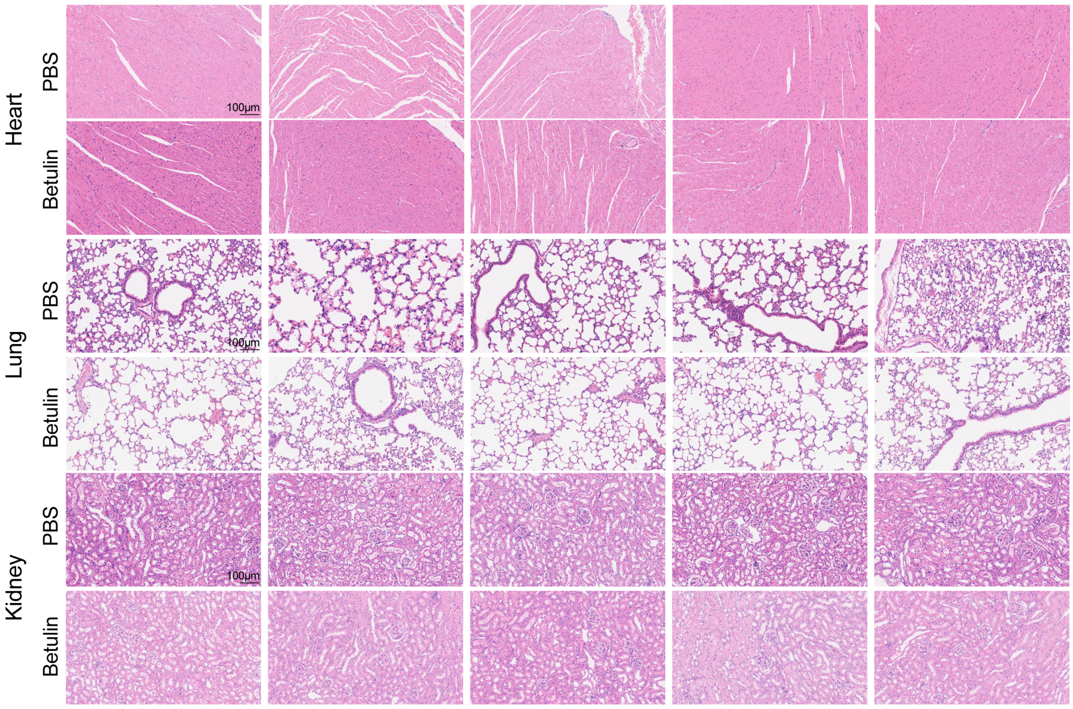

Figure S7. Representative images of HE staining in heart, lung and kidney tissue sections of mice.

Table S1. The sequences of the primers used in this study.

Names	Sequences
GAPDH-primer-F	GGAGCGAGATCCCTCCAAAAT
GAPDH-primer-R	GGCTGTTGTCATACTTCTCATGG
PDGFC-primer-F	GACTCAGGCGGAATCCAACC
PDGFC-primer-R	CTTGGGCTGTGAATACTTCCATT
SREBF1-primer-F	ACAGTGACTTCCCTGGCCTAT
SREBF1-primer-R	GCATGGACGGGTACATCTTCAA
ACLY-primer-F	TCGGCCAAGGCAATTTCAGAG
ACLY-primer-R	CGAGCATACTTGAACCGATTCT
FASN-primer-F	AAGGACCTGTCTAGGTTTGATGC
FASN-primer-R	TGGCTTCATAGGTGACTTCCA
SCD-primer-F	TCTAGCTCCTATACCACCACCA
SCD-primer-R	TCGTCTCCAACTTATCTCCTCC
ACSS2-primer-F	AAAGGAGCAACTACCAACATCTG
ACSS2-primer-R	GCTGAACTGACACACTTGGAC
ACACA-primer-F	ATGTCTGGCTTGCACCTAGTA
ACACA-primer-R	CCCCAAAGCGAGTAACAAATTCT
ELOVL6-primer-F	AACGAGCAAAGTTTGAACTGAGG
ELOVL6-primer-R	TCGAAGAGCACCGAATATACTGA
